# Host species adaptation of TLR5 signalling and flagellin recognition

**DOI:** 10.1038/s41598-017-17935-5

**Published:** 2017-12-15

**Authors:** Amin Tahoun, Kirsty Jensen, Yolanda Corripio-Miyar, Sean McAteer, David G. E. Smith, Tom N. McNeilly, David L. Gally, Elizabeth J. Glass

**Affiliations:** 1 Division of Immunity and Infection, The Roslin Institute and R(D)SVS, The University of Edinburgh, Easter Bush, Midlothian, EH25 9RG UK; 2 Moredun Research Institute, Pentlands Science Park, Bush Loan, Penicuik, EH26 OPZ UK; 3 University of Glasgow, Glasgow, G12 8TA UK; 4 Present Address: Faculty of Veterinary Medicine, Kafrelsheikh University, 33516 Kafr el-Sheikh, Egypt; 5 Present Address: Moredun Research Institute, Pentlands Science Park, Bush Loan, Penicuik, EH26 OPZ UK; 6 Present Address: Institute of Biological Chemistry, Biophysics and Bioengineering, Heriot Watt University, Edinburgh, EH14 4AS UK

## Abstract

Toll-like receptor 5 (TLR5) recognition of flagellin instigates inflammatory signalling. Significant sequence variation in TLR5 exists between animal species but its impact on activity is less well understood. Building on our previous research that bovine TLR5 (bTLR5) is functional, we compared human and bovine TLR5 activity and signalling in cognate cell lines. bTLR5 induced higher levels of CXCL8 when expressed in bovine cells and reciprocal results were found for human TLR5 (hTLR5) in human cells, indicative of host cell specificity in this response. Analysis of Toll/interleukin-1 receptor (TIR) sequences indicated that these differential responses involve cognate MyD88 recognition. siRNA knockdowns and inhibitor experiments demonstrated that there are some host differences in signalling. Although, PI3K activation is required for bTLR5 signalling, mutating bTLR5 F798 to hTLR5 Y798 within a putative PI3K motif resulted in a significantly reduced response. All ruminants have F798 in contrast to most other species, suggesting that TLR5 signalling has evolved differently in ruminants. Evolutionary divergence between bovine and human TLR5 was also apparent in relation to responses measured to diverse bacterial flagellins. Our results underscore the importance of species specific studies and how differences may alter efficacy of TLR-based vaccine adjuvants.

## Introduction

Toll-like receptors (TLRs) are the most studied of the key pattern recognition receptors (PRRs) in multicellular eukaryotic hosts that function to recognise pathogen invasion and signal danger, leading to cascades of defensive responses (reviewed by 1). Mammals have between 1 and 13 distinct TLRs which are specific for various molecules expressed by pathogens^1^. TLRs are membrane-bound proteins mainly expressed in compartments that are entryways for invading pathogens such as the cell surface or endosome of innate immune cells and epithelial cells. Each TLR contains a ligand binding ecto-domain as well as an intracellular Toll-Interleukin receptor domain (TIR). Ligand binding results in conformational changes in the TLR and triggers signalling cascades involving interactions between the TIR domain and various adapters, for example MyD88, which can also possess a TIR domain^1^.

Although TLRs are relatively well conserved between species, in recent years it has become evident that there are potentially functionally important species differences in TLRs in both the ligand binding and TIR domains^2^. Non-synonymous amino acid substitutions and polymorphisms potentially reflect adaptations as a result of exposure to distinct pathogens during the life history of particular hosts^3,4^. These differences may underlie the varying way in which different host species respond to pathogens and have significant consequences for predicting the outcomes of infection and vaccination^5^. Thus the use of TLR ligands as potent adjuvants in vaccines may require modification according to species-specific sequence differences in key residues in TLR molecules.

TLR5 is the only TLR that recognises a protein ligand, flagellin which forms the core of the main motility organelle of flagellated bacteria and consequently flagellin constructs have been explored as potential vaccine components in various host species including humans^6^ and cattle^7^. Our earlier studies had indicated that bovine TLR5 contains a number of positively selected sites in the extracellular and intracellular domains^2,7^. We also have also recently shown that contrary to some reports^8,9^, bovine TLR5 (bTLR5) is functional in both human (HEK293) and bovine epithelial cell lines (EBL), as well as in bovine macrophages. Signalling through bTLR5 with H7 flagellin ligand derived from *E. coli* O157 resulted in NFκB reporter activation and up-regulation of CXCL8 mRNA in these cells as well as secretion of the chemokine^7^. In addition, TLR5-specific knock-down using siRNA significantly reduced the response to flagellin in bovine macrophages^7^. However, it was noted that in a human cell line (HEK cells), at low concentrations of flagellin, human TLR5 (hTLR5) was somewhat more responsive than bTLR5, indicating that amino acid differences between bovine and human TLR5 may result in differences in flagellin binding and/or signalling^7^.

The major binding interaction between TLR5 and flagellin was identified as TLR5 leucine rich repeat (LRR)9 with the flagellin D1 domain, especially the highly conserved R90 residue^10^. Mutation of R90 to other residues abrogates activation of both human TLR5^11^ as well as bovine TLR5^7^, confirming the importance of the LRR9 region across a wide range of species. Furthermore cattle immunised with *E. coli* O157 H7 flagellin consistently had lower humoral responses with R90 mutated to T90 compared to the wild-type flagellin^7^.

In the current study, we sub-cloned the TLR5 of both species into a modified ptGFP1 vector^12^ and transfected these constructs into both HEK293T (human) and EBL (bovine) cell lines. This ensured that a high percentage of cells (90–95%) expressed the constructs to enable valid comparisons in responses induced by the TLRs and in different host cell backgrounds. We then explored the signalling pathways utilised by the TLR5 variants in both their cognate and distinct host backgrounds as well as their relative responsiveness to flagellins purified from different bacterial genera.

## Results

### Comparison of human and human TLR5 signalling in cells lines from the two different hosts

**Figure 1:**
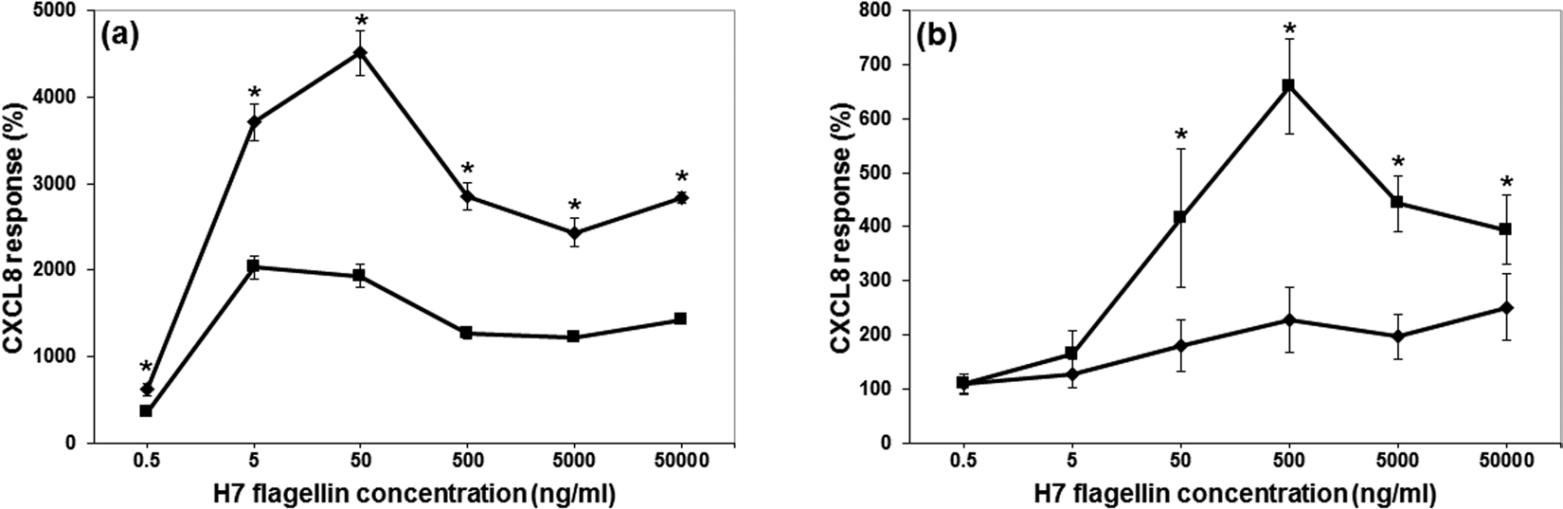
The activity of bovine and human TLR5 differs with cell background. CXCL8 protein release from (**a**) HEK293T cells and (**b**) EBL cells stably transfected with human TLR5 (♦) or bovine TLR5 (■), activated with *E. coli* H7 flagellin at various concentrations. The Figures show the average CXCL8 protein response for each H7 concentration as a percentage of the background response in non-transfected cells. The error bars illustrate the standard error of the mean for three experiments, each with three technical replicates. The average CXCL8 protein release in response to flagellin was significantly different in cells expressing bovine or human forms of TLR5 by ANOVA (P < 0.001) and * denotes which doses were significant by subsequent Tukey’s test (P < 0.05).

From previous published research, it was evident that when using lower levels of H7 flagellin, HEK cells transfected with bovine TLR5 were less responsive when transfected with human TLR5^7^. While this may be due to functional differences in TLR5 activity, at the time we could not rule out that it may be due to lower expression levels of bovine TLR5. To our knowledge, there is no commercial anti-human TLR5 monoclonal or polyclonal antibody that also detects bovine TLR5. As an alternative, the required TLR was expressed in tandem with GFP using a modified ptGFP vector (Materials and Methods)^7^. GFP positive transfected cells were sorted to high GFP positivity. These cells also had similar mean fluorescent intensities (MFI) (Supplementary Fig. S1). Using this approach, HEK-293T or EBL were transfected with either the human or the bovine TLR5 clone, which enabled us to directly compare their responses to H7 flagella (Fig. 1a–d). In the human HEK background the bovine TLR5 had a significantly reduced response (p < 0.001) to H7 flagellin and post-hoc analysis indicated that this was at every dose (0.5 ng/ml to 50,000 ng/ml) of H7 compared to the human TLR5 (p < 0.05 for each dose) (Fig. 1(a)). By contrast, the complete reversal of this occurred when the same human and bovine TLR5 GFP clones were transfected into a bovine lung epithelial cell line (EBL) (Fig. 1(b)). In this case, the bTLR5 was significantly more responsive to H7 (p < 0.001) and across a range of H7 concentrations, from 50 ng/ml to 50,000 ng/ml, compared to hTLR5 (p < 0.05 for each dose) (Fig. 1B). (In Supplementary Fig. S2 the levels of CXCL8 in pg/ml are shown for each condition, along with the background levels for the non-transfected cells).

### Site specific mutation of bovine TLR5

**Figure 2:**
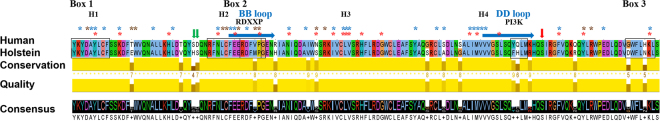
Comparison of human and bovine sequence alignment of the TIR domain of TLR5. Potentially functionally relevant positions are shown (using clustal omega and displayed using Jalview). The position of Boxes 1–3^42^, RDXXP motif^15^ and putative PI3 kinase motif^2^ are shown as black outlines; red vertical arrow indicates the partially conserved S805 that may be phosphorylated by PKD and required for signalling^43^; BB loop and DD loop^42^ are indicated by the horizontal blue arrows; red stars indicate similar or conserved amino acids across TIR domains of animals, plants and bacteria^14^; brown stars are totally conserved and blue stars are semi-conserved residues across TLR TIR domains^13^ and H1–4 indicate positions that have been identified as conserved hubs of communication between ectodomain and TIR domain following ligand binding^13^; the double green vertical arrows indicate: H720 and S721 identified as positively selected^2,16^, respectively.

Since we had, as far as possible, controlled for expression levels of each TLR5 in the two transfected cell lines, we interpret the data to show that there is a host species incompatibility in the interaction of TLR5 and its intracellular partners, leading to differences in signalling. To examine this, we compared the amino acid sequence of the intracellular signalling TIR domain of human and bovine TLR5 (Fig. 2). As there are now additional artiodactyla and ruminant TLR5 sequences available, compared to the analysis in our earlier paper^2^ we re-analysed this new data-set (Supplementary Table S1) focusing on a comparison with human and other vertebrate species (Supplementary Fig. S3). The TIR region of TLR5 is extremely highly conserved across species compared to the extracellular domain. Across TIR domain proteins, a set of motifs and conserved residues have been identified although their functional significance remains unclear^13–15^ with the majority not having been formally tested for TLR5. In general, the various motifs and potential intra-domain and inter-domain interaction motifs are conserved across the TLR5 sequences with very few changes in the putative functional sites (Fig. 2; Supplementary Fig. S3).

However, there are three exceptions that may underlie functional differences in signalling between cattle and human TLR5 molecules (Fig. 2; Supplementary Fig. S3). First, the additional ruminant and cetartiodactyla sequences now available indicate that of two amino acids previously identified as positively selected^2,16^, H720 is a *Bos*-specific change and S721 is a *Ruminantia-*specific change. Second, a motif within the BB loop, RDXXP which likely interacts with MyD88^15^, has a *Ruminantia-*specific change to RDFMP which is distinct from all other species where the motif is RDFI/V/LP (Supplementary Fig. S3). Third, two contiguous amino acids that are contained in a putative PI3K motif^17^ within the DD loop are clearly distinct in ruminants being “FH” whereas in almost all other species, including human, the motif is “YQ” (Supplementary Fig. S3). Osvaldova and colleagues^9^ suggested that this difference resulted in an inability of bTLR5 to signal, and replaced FH for YQ in bTLR5 with partial restoration of its activity in HEK cells. However, our data above demonstrates that wild-type bTLR5 is able to signal in both a human and bovine cell background, despite the lack of this putative PI3K motif.

**Figure 3:**
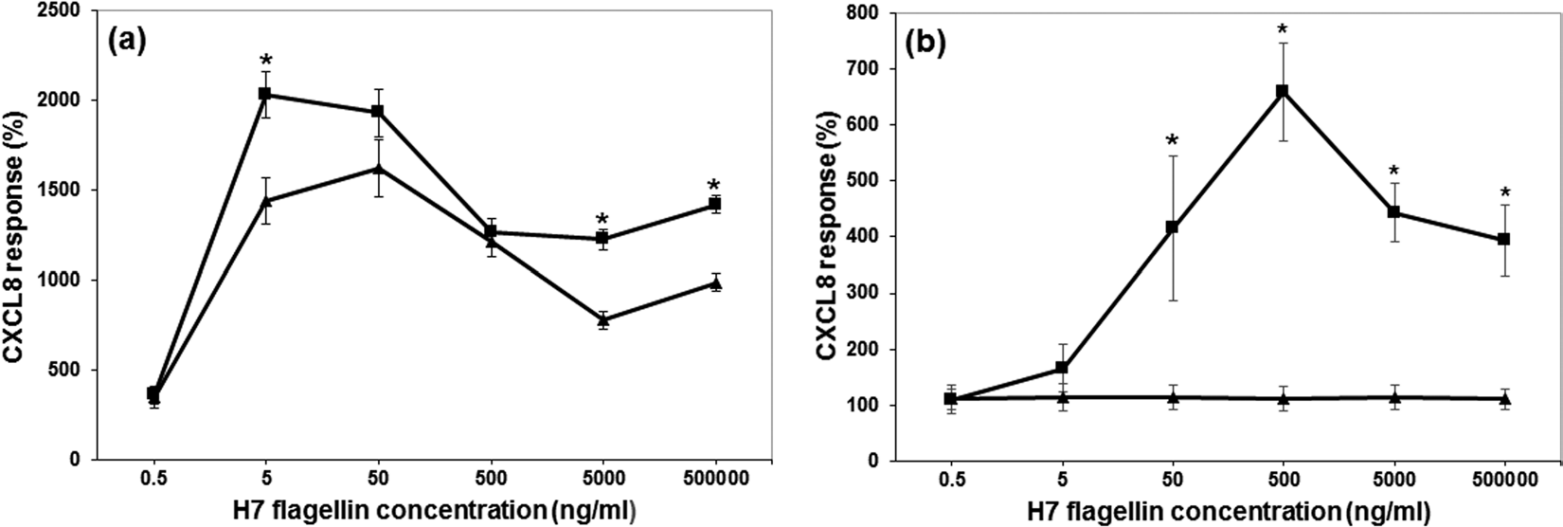
The effect of a mutation in the cytosolic region of bovine TLR5 differs with cell background. CXCL8 protein release was measured in (**a**) HEK293T cells or (**b**) EBL cells stably transfected with wild-type bovine TLR5 (■) or F798Y mutant (▲) after stimulation with 0.5–50,000 ng/ml *E. coli* H7 flagellin. The graph shows the average protein response as a percentage of the background response in non-transfected cells. The error bars illustrate the standard error of the mean of three experiments, each with three technical replicates. The average CXCL8 protein release in response to flagellin was significantly different in cells expressing bovine wild-type or mutant bTLR5F798Y forms of TLR5 by ANOVA (P < 0.001) and * denotes which doses were significant by subsequent Tukey’s test (P < 0.05).

Given the conflicting data between our study and that of Osvaldova *et al*.^9^, we initially exchanged the bTLR5 amino acid F at position 798 with Y as present in the hTLR5. We investigated the response of the mutated bTLR5 (bTLR5F798Y) and compared its activity with the wild-type bTLR5 in both human HEK and bovine EBL cells (Fig. 3(a) and (b)). The same GFP-containing vector background and cell sorting were used to achieve high expression levels of the bTLR5F798Y variant (Supplementary Fig. S1). There was a significantly lower response to flagellin for the bovine mutant bTLR5F798Y compared to bovine wild-type TLR5 H7 (p < 0.001) in HEK cells (Fig. 3(a); Supplementary Fig. S4(a)). Significantly and in contrast to the result in the HEK cells, bTLR5F798Y expression in EBLs resulted in substantially ablated responses to H7 compared to the wild type molecule and was indistinguishable from the response by untransfected cells (P < 0.001) (Fig. 3(b); Supplementary Fig. S4(b)).

### The role of PI3K signalling in host specific TLR5 signalling

**Figure 4:**
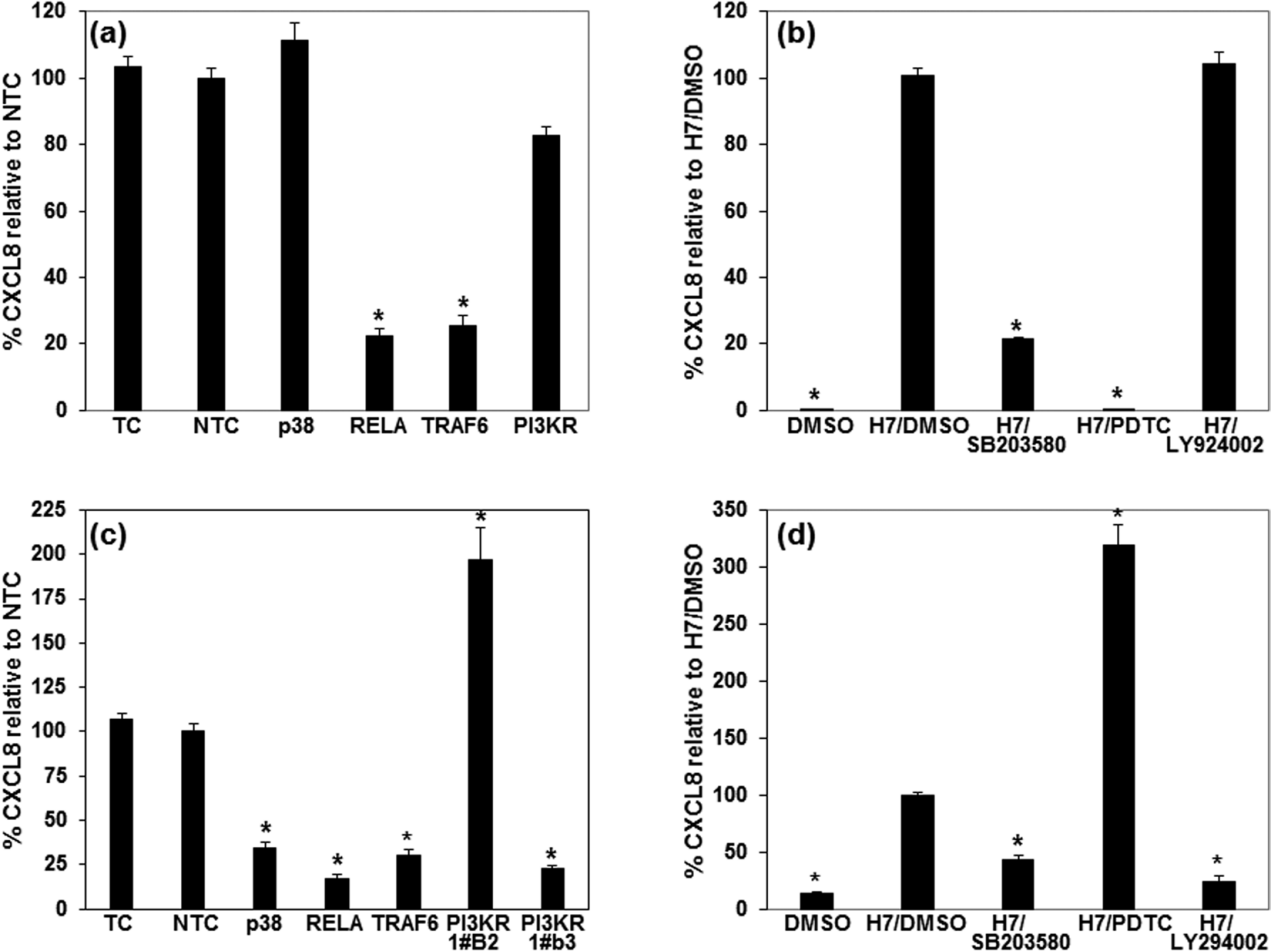
Blocking TLR signalling with down-stream inhibitors and targeted siRNA reveals similarities and differences between human and bovine signalling pathways. HEK293T cells stably transfected with human TLR5 (**a**) and EBL cells stably transfected with bovine TLR5 (**c**) were transiently transfected with siRNA for p38, RELA, TRAF6 and PIK3R1. PIK3R1 (human) and PIK3R1#b3 (bovine) are predicted to recognize all splice variants, thereby reducing levels of all three regulatory subunits of class IA PI3K encoded by human or bovine *PIK3R1* respectively; PIK3R1#b2 (bovine) is predicted to only recognize the splice variant encoding the p85A regulatory subunit. In addition, cells were treated with transfection reagent only (TC) or transfected with non-target control siRNA (NTC). After 24hr cells were activated with 100 ng/ml *E. coli* H7 flagellin. The graphs illustrates the average release of CXCL8 protein relative to that released by NTC samples. The error bars illustrate the standard error of the mean of three experiments, each with three technical replicates. HEK293T cells stably transfected with human TLR5 (**b**) and EBL cells stably transfected with bovine TLR5 (**d**) were treated with inhibitors of p38 (5 μM SB203580), NF-κB (100 μM PDTC) and PI3K (50 μM LY294002) or DMSO before stimulation with ng/ml *E. coli* H7 flagellin. The graphs illustrates the average release of CXCL8 protein relative to that released by DMSO treated samples. The error bars illustrate the standard error of the mean of three experiments, each with three technical replicates. *Denotes that the average CXCL8 level was statistically significantly different from (**a** and **c**) NTC or (**b** and **d**) DMSO and H7 treated samples by ANOVA and subsequent Tukey’s test (P < 0.05).

We then investigated the role of PIK3RI regulatory subunit of PI3K in TLR5 signalling using both siRNA and a PI3K inhibitor comparing hTLR5 responses in HEK cells with bTLR5 responses in EBL. The CXCL8 response induced by interaction of hTLR5 with H7 flagellin was reduced but not significantly by siRNA for PIK3R1, which knocks down expression of all three regulatory subunits of class IA PI3K encoded by the human *PIK3R1* gene: p85A, p55A and p50A (Fig. 4(a)). Furthermore, no inhibition was observed when the PI3K inhibitor LY294002 was added to the interaction (Fig. 4(b)). In contrast, siRNAs designed to different regions of the bovine *PIK3R1* gene had different effects on the CXCL8 response driven by flagellin activation of bTLR5 expressed in EBL cells (Fig. 4(c)). PIK3R1#b3 resulted in significant inhibition of the response, whereas PIK3R1#b2 resulted in enhancement of the CXCL8 response. PIK3R1#b3 is predicted to recognize all splice variants, thereby reducing levels of all three regulatory subunits encoded by *PIK3R1*, and is therefore comparable to the siRNA against human PIK3R1. In contrast, PIK3R1#b2 is predicted to only recognize the splice variant encoding the p85A regulatory subunit. The inhibitor, LY294002 in contrast to the effects on hTLR5 activation, significantly ablated the response of bTLR5 to H7 flagellin (Fig. 4(d)), in agreement with the result for PIK3R1#b3. This data demonstrates that TLR5 activation by bTLR5 in a cognate bovine cell line requires PIK3, whereas there was no evidence for this in the parallel human system.

### Comparative analysis of TRAF6, RELA and p38 in host specific TLR5 signalling

We also investigated the role of TRAF6, RELA subunit of NFκB and p38 using siRNA knock-down and specific inhibitors. As expected, RELA knock-down and the presence of PDTC, an inhibitor of NFκB, resulted in substantial reduction of CXCL8 production in response to H7 flagellin by hTLR5 in the HEK background (p < 0.05) (Fig. 4(a) and (b) respectively). Similar findings were obtained for bTLR5 in EBL with RELA siRNA leading to ablation of the CXCL8 response (p < 0.05) (Fig. 4(c)). However, in contrast, the inhibitor PDTC resulted in enhanced release of CXCL8 in the bovine combination (Fig. 4(d)). Knock-down of TRAF6 resulted in similar reduction in CXCL8 production in response to hTLR5 in HEK or bTLR5 in EBL (Fig. 4(a) and (c) respectively). For the p38 pathway we observed further discrepancies between the siRNA knock-down and inhibitors. p38 siRNA had no effect on the CXCL8 response of hTLR5 in HEK (Fig. 4(a)) whereas the presence of the p38 inhibitor, SB203580, led to a significant reduction in activity (Fig. 4(b)). However, both siRNA for p38 and its inhibitor reduced the response of bTLR5 to H7 flagellin in EBL (Fig. 4(c) and (d)). These results indicate that hTLR5 and bTLR5 both signal through TRAF6 to the NFκB and p38 dependent pathways, albeit with some potential differences.

### Differential activation of host-specific TLRs by flagellin preparations from different bacterial genera

Our earlier studies^2,7^ had also indicated that bovine TLR5 contains a number of positively selected sites in the extracellular domain which might also account for functional differences between bTLR5 and hTLR5. Table 1 compares the key regions in the ectodomain of human, bovine and zebrafish TLR5. These are the main flagellin binding regions: LRRNT-LRR6 (interface A) which bind to the C-terminal half of flagellin D1 domain and LRR7-LRR10 (interface B) which bind to the N-terminal half of the flagellin D1 domain. A total of thirty-three amino acids within both interfaces of zebrafish TLR5 were identified as interacting with flagellin. Of the five amino acids that are conserved across all three species (S6, G266, F269, N273 and K275 (numbered as for bovine TLR5)), four are within the major binding region, LRR9^10^, highlighting the major importance of this LRR. We re-analysed our artiodactyla-focused data-set of TLR5 sequences (Supplementary Table S1) comparing these to human and other vertebrate species (Supplementary Fig. S5). Six of the thirty-three TLR5 binding residues were previously identified as under positive selection^2,16^ (Table 1). The new data reveals that five of these are restricted to *Ruminantia* (L34, G104, G239, S268 and S272), with the latter two residues also within LRR9 (Table 1; Supplementary Fig. S5). Q207 is restricted to *Bos* species, and all other ruminants have Histidine at this position whereas most other species express different residues. In addition, a further four TLR5 binding amino acids are clearly associated with *Bos* and *Bison* species (S128, F179, K181 and R261) or with the *Ruminantia* clade (Y297). Taken together, these host specific differences between human and bovine TLR5 could result in differing interactions with flagellins that vary in their TLR5 interacting primary sequences within the D1 domain of the flagellin subunit.

**Table 1:** Comparison of amino acids involved in binding between human, bovine & zebrafish TLR5 & flagellins.

TLR5 Primary binding interface-A/flagellin D1-Ct									TLR5 Primary binding interface-B/flagellin D1-Nt								
LRR	TLR5			Flagellins					LRR	TLR5			Flagellins				
	**Bov**	**Hum**	**Zb**	**S.D. FliC**	**S.T FliC**	**E.c H7**	**L.i FlaA**	**B.t FlaA**		**Bov**	**Hum**	**Zb**	**S.D FliC**	**S.T FliC**	**E.c H7**	**L.i FlaA**	**B.t FlaA**
LRRNT	I32	F32	I33	N438	N	N	N	T	LRR7	Q207*	S207	T208	Q89	Q	Q	Q	Q
LRRNT	**L34** ^1^	R34	I35	S434, T437, N438	S,T, N	S, T N	Y, S N	A, T T	LRR7	Y209	Y209	Q210*	D412^4^, R92*	D, R	D, R	D, R	D, R
LRRNT	**S35**	F35	N36	E153^2^, A435	E, A	E, A	D, N	Q, I	LRR7	—	—	N213	E93	E	E	Q	Q
LRRNT	C36	C36	R37*	N438*	N	N	N	T	LRR7	V212	V212	Y215	V96, Q97, N100, **G101** ^6^	V, Q, N, S	V, Q,T, G	V, Q, N, G	V, Q, N, G
LRR1	L52	L52	D53	T437	T	T	S	T	LRR8	**G239**	T239	K242*	E93, Q97*	E, Q	E, Q	Q, Q	Q, Q
LRR1	*S54*	*S54*	*S55*	S434	S	S	Y	A	**LRR9**	**R261**	H261	N265	N82, N86	N, N	S, N	S, S	T, N
LRR1	F55	F55	L56	R431, S434	R, S	R, S	R, Y	R, A	**LRR9**	H263	H263	Y267^†^	N86, Q89, R90*	N, Q, R	N, Q, R	S, Q, R	N, Q, R
LRR2	E76	E76	K77	T437	T	T	S	T	**LRR9**	I264	I264	N268	**R90**, E93	R, E	R, E	R, Q	R, Q
LRR2	G78	G78	E79*	N430*	N	N	S	N	**LRR9**	*G266*	*G266*	*G270* ^††^	R90**	R	R	R	R
LRR2	T79	S79	Q80**	A427, N430*, R431*	A, N, R	A, N, R	A, S, R	A, N, R	**LRR9**	S267^5^	A267	S271^†^	R90*	R	R	R	R
LRR3	**G104**	S104	Y105	G426, A427, N430	G, A, N	G, A, N	G, A, S	G, A, N	**LRR9**	**S268**	G268	S272*	R118*	R	R	L	Q
LRR4	**S128**	F128	Q129*	S423^†^, A427	S, A	S, A	A, A	A, A	**LRR9**	*F269*	*F269*	*F273*	Q117	Q	S	S	Q
LRR5	K154	K154	D155**	R422**, S423	R, S	R, S	R, A	Q, A	**LRR9**	F271	F271	H275*	**D113**, E114, Q117*	A, E, Q	D, E, S	A, E, S	Q, E, Q
LRR6	**F179**	S179	F180	E78^3^, R422	E, R	E, R	D, R	S, Q	**LRR9**	**S272** ^7^	H272	T276	S110	S	S	Q	A
LRR6	**K181**	Q181	K182*	D419*	D	D	S	N	**LRR9**	*N273*	*N273*	*N277* ^†^ ***	L94, Q97*, S110^†^, I111^†^, E114*	L, Q, S, I, E	L, Q, S, I, E	L, Q, Q, Y, E	L, Q, A, L, E
									**LRR9**	L274	I274	F278	**R90**, E93, L94, Q97	R, E, L, Q	R, E, L, Q	R, Q, L, Q	R, Q, L, Q
									**LRR9**	*K275*	*K275*	*K279*	Q97	Q	Q	Q	Q
									LRR10	**Y297**	F297	K303	N87, **R90**, R118	N, R, R	N, R, R	I, R, L	S, R, Q

Bov = bovine; Hum = human; Zb = zebrafish; S.D. = *Salmonella enterica* serovar Dublin; S.T. = *Salmonella enterica* serovar Typhimurium; E.c. = *E. coli*; L.i. = *Listeria ivanovii*; B.t. = *Burkholderia thailandensis*.

Flagellin amino acid numbering according to Smith *et al*. (2003)^11^; TLR5 amino acid numbering: for Zb, according to Yoon *et al*. (2012)^10^; for Bov & Hum, according to Smith *et al*. (2012)^2^.

**Figure 5:**
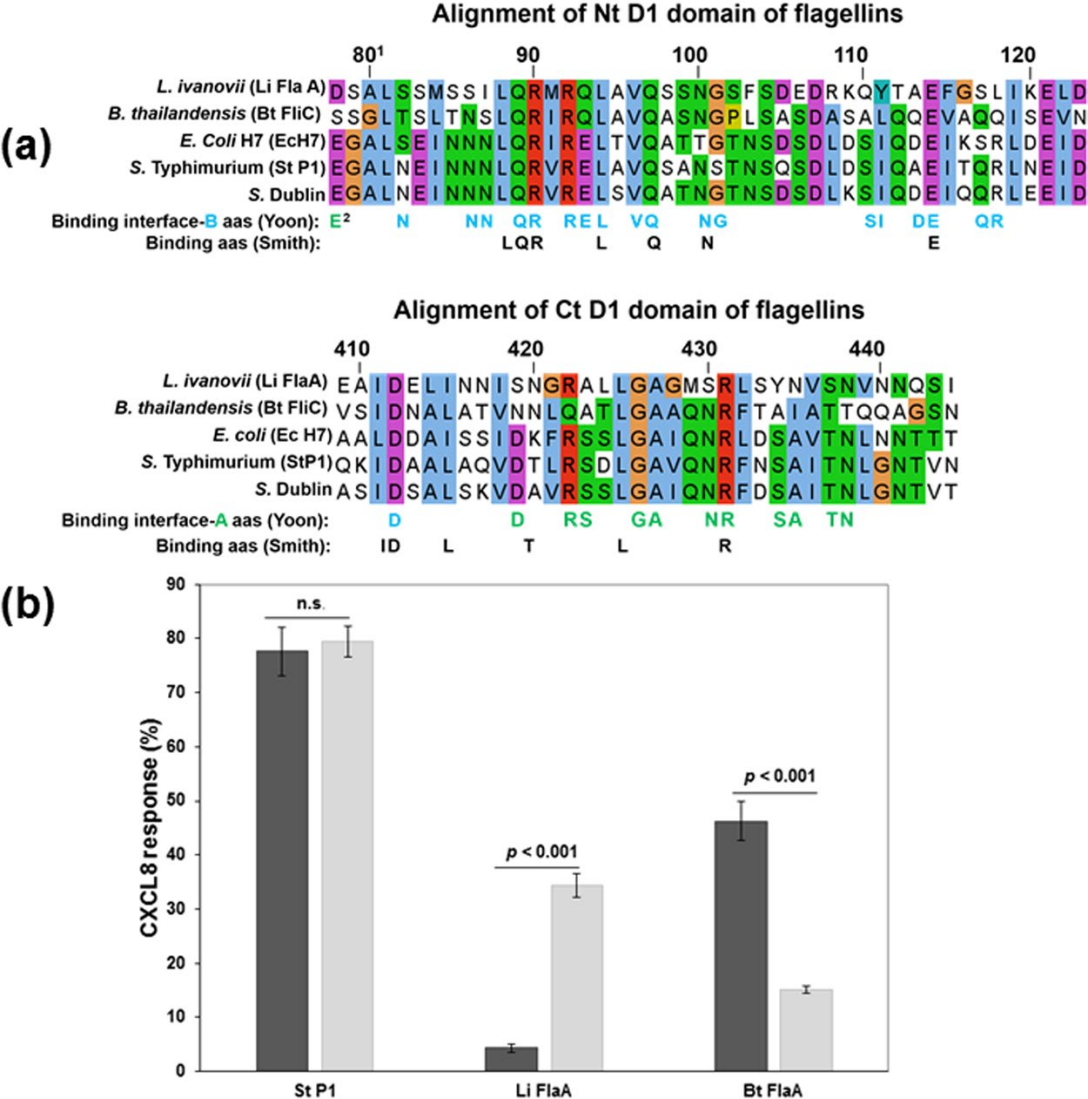
Diverse flagellin alignments of Nt and Ct D1 domains, indicating identified TLR5 binding amino acids and relative responses to these by bovine and human TLR5. (**a**) ^1^Numbering according to Smith *et al*. (2003)^11^. ^2^Blue amino acids bind to TLR5 binding interface-B; green amino-acids bind to TLR5 binding interface-A, according to Yoon *et al*. (2012)^10^; Black amino acids bind to TLR5 according to Smith *et al*. (2003)^11^. aas: amino acids. (**b**) The relative CXCL8 protein release from HEK293T cells stably transfected with human TLR5 and EBL cells stably transfected with bovine TLR5 after stimulation with 50ng/ml *S*. Typhimurium P1 (St P1), *Listeria ivanovii* FlaA (Li FlaA) and *Burkholderia thailandensis* FliC (Bt FliC) compared to the response to *E. coli* H7 (Ec H7). Error bars illustrate the standard errors of the mean for three experiments, each with three technical replicates. *Denotes that the average CXCL8 protein release in response to the different flagellin was significantly different by ANOVA and subsequent Tukey’s test (P < 0.05).

^1^Positive selection detected in ***Bos*** or ***Ruminantia*** clades^1,2^, or associated with these species (this paper) is shown in bold; Conserved TLR5 amino acids across host species shown in italic; R90 is completely conserved across all five flagellins and is shown in bold-underline; ^2^Not shown in Fig. 5 as is located in the non D1 domains of flagellins; ^3^interface-A/Flagellin D1-Nt interaction; ^4^interface-B/Flagellin D1-Ct interaction; ^5^P in mice is sufficient to account for species specific binding^33^ (≡P268 in their paper) & alters LRR9 loop structure compared to human^25^; ^6^Flagellin amino acids shown in bold are not conserved between *Salmonella* Dublin and *S*. Typhimurium; ^7^Q in mice is sufficient to alter the shape of the LRR9 loop compared to human LRR9 loop^19^.

*H-bond or ^†^ salt bridge interactions that involve a side chain (multiple * or ^†^ reflect the number of H-bonds/salt bridges) according to Yoon *et al*. (2012)^10^.

In order to explore this further, we compared the sequence of the two TLR5 binding domains (Nt D1 and Ct D1) of *E. coli* H7 flagellin with the equivalent sequences of specific flagellins that encompass variations in their TLR5 binding domains (Fig. 5(a)). These included *S*. Dublin, *S*. Typhimurium, *L. ivanovii* and *B. thailandensis* flagellins. The flagellins from *L. ivanovii* and *B. thailandensis* are quite distinct from each other and from those expressed by both *Salmonella* and *E. coli* which are closely related irrespective of the serogroup selected (Table 1 and Fig. 5(a)). Eleven of the 31 amino acids identified by Yoon *et al*.^10^ as important for *S*. Dublin flagellin recognition, are conserved between all five flagellins including R90, E114 and Q97 (Fig. 5(a)) These three amino acids make 9 interactions with the major LRR9 TLR5 binding site, the majority of which are to highly conserved TLR5 amino acids (Table 1). Indeed, in total, the conserved amino acids in flagellins mainly interact with conserved TLR5 amino acids (Table 1 and Fig. 5(a)).

However, there are a few differences between these flagellins in their binding amino acids that coincide with differences between human and bovine binding amino acids. For example R118 in *E. coli* and *Salmonella* is exchanged with Q118 and L118 in *B. thailandensis* and *L. ivanovii* Nt D1 flagellin domain, and is reflected in ruminant specific substitutions in bTLR5: S268 and Y297 in LRR9 and LRR10, compared to hTLR5: G268 and F297 (Fig. 5(a), Table 1, Supplementary Fig. S5).

In order to test whether any of the differences in the human and bovine TLR5 ectodomains resulted in differences in the way they interacted with these flagellins, we compared the relative CXCL8 responses of hTLR5 in HEK cells with bTLR5 in EBL cells to flagellin preparations from these different bacterial species. The relative responses of hTLR5 and bTLR5 to three of these flagellin preparations were then compared directly to their *E. coli* (H7) responses (Fig. 5(b)). There was no significant difference in their relative responses to *S*. Typhimurium flagellins. In contrast, there was a significant reversal in the relative responses of hTLR5 and bTLR5 to the more diverse flagellins isolated from *L. ivanovii* and *B. thailandensis* (p < 0.001).

## Discussion

TLRs are fundamental components of the innate response and understanding variation in pattern recognition and signalling by related TLRs in different host species provides insights into differences in infection pressure that drive their evolution. This study has analysed the functional differences between human and bovine TLR5 homologues and reaches two significant conclusions^1^: that these TLR5s do not use identical signalling pathways in their respective hosts to induce inflammation and^2^ that the two TLR5s differ in their interactions with flagellin. Taken together our data suggest that effective TLR5 signalling in each species reflects co-evolution of the TLR5 molecules with the repertoire of flagellated bacterial pathogens that have affected their hosts, as well as with the TLR5-adaptor proteins and signalling components. These observations have important implications for the application of immune-modulators, signalling inhibitors and PAMP-based adjuvants for use in animals especially in terms of interpretation of data from experimental studies in mice and humans.

There have been several reports of species specific differences in TLR5 which result in functional changes^2,7–9,18–20^; most of these papers have used human cell lines to investigate host species specificity. We have shown that bovine TLR5, in contrast to some earlier reports^8,9^, is fully functional in both a bovine and human cell line background, as well as in bovine macrophages and, importantly, is active *in vivo* ^7^. However, we could not rule out the possibility that some of the down-stream differences between hTLR5 and bTLR5 were a result of differences in expression in the cell lines. As a suitable anti-bTLR5 antibody was not available, we used a modified ptGFP1 vector^12^ which enabled us to flow sort transfected cell lines to high purity, with the majority of cells expressing hTLR5 or bTLR5 at similar fluorescence levels. Although we cannot categorically rule out that this approach may have not resulted in equivalent numbers of molecules of hTLR5 and bTLR5 at the cell surface, the proportion of transfected cells expressing each TLR5 was generally greater than 90% GFP positive with similar MFI levels. Furthermore, the clear differences in the dose response curves for each species’ TLR5 between the human and bovine cell lines strongly suggest that the differences described in this paper reflect differences in TLR5 biology between hTLR5 and bTLR5, rather than differences in levels of cell surface TLR5. Additionally, by transfecting each TLR5 into both cell lines with different host backgrounds, it was possible to explore the effect of host species differences in the extracellular domains separately from differences in the TIR region.

Stronger responses to flagellin were observed with hTLR5 than bTLR5 in cells with a human background whereas in contrast, the identical bTLR5 construct was activated to a significantly higher level in EBLs than was hTLR5. This suggested that the optimum interaction between TLR5 and its adapters and down-stream signalling molecules may be species dependent. Until now, this has not been considered, partly because there is higher conservation between the transmembrane and TIR region sequences and also different species’ TLR5 constructs have shown functional activity in human cell lines, even those that are quite evolutionarily distinct from mammals such as chicken and reptile TLR5^18,20^. Our data would suggest that this approach may mask important differences in signalling as well as flagellin binding. Thus studies that compare TLR activity and specificity between species may require cells of the appropriate species, as otherwise erroneous conclusions may be drawn.

In general, the TIR domain of proteins contains highly conserved loops and motifs (boxes) believed to play a role in recruiting adapters and in dimerisation of TLR monomers following ligand binding^21^. Several TIR domain structures have been described and some have been crystallised (summarised by Ve and colleagues^14^). The loops are very close to regions that are highly conserved among all TLRs and between species and may act as part of an evolutionarily conserved interaction network between different TIR containing proteins^13^. However, conformational differences between different TIR domain containing proteins exist, and two of the so-called conserved motifs (boxes 2 and 3) are in fact not that well conserved, with evidence that other regions may also play functionally important roles^14^.

As with most other TLRs, TLR5 first interacts with the IL-1R/TIR containing adapter MyD88^21^ which couples the cell surface TLR5 to down-stream intracellular signalling pathways through TRAF6^22^. This results in the activation of transcription factors such as NFκB and the MAPK pathways including p38 which leads to the production of pro-inflammatory cytokines, such as CXCL8^23^. However, there have been relatively few papers that detail the structure-function relationships between the TIR domain of TLR5 and its downstream partners, and even fewer that have investigated these interactions in species other than man and mouse. By analogy with other better studied TLRs, it is likely that TLR5-MyD88 interaction occurs at least in part through the BB and DD loops within the TIR domains of TLR5 and MyD88. However, TIR domain loops are divergent among different TLRs and these differences play a role in the specific outcomes of TLR engagement with ligands. The BB loop in TLR5 contains a highly conserved motif RDxϕ_1_ϕ_2_G with a conserved Pro at ϕ_2_ which occupies the tip of the loop and is essential for TLR5 activity^24^. However the TLR5 BB loop motif has one amino acid change between cow and human (RDFMP and RDFVP respectively) which has been conserved across *Ruminantia* species and may therefore be functionally relevant. Evidence has indicated that the BB motif in other TLRs interacts with a binding site (BS III) in MyD88 near the N-terminus^15^. The core residues in MyD88 BS III, W284, R288 and K291, are conserved between bovine MyD88 and human MyD88 (data not shown). However bMyD88 differs by two amino acids at the start of this site from the human form of MyD88, being Q-N rather than K283-S284. These changes are again *Ruminantia*-specific, suggesting that they may have a functional consequence. Further work is needed to confirm whether or not these changes are at least in part responsible for the differences observed in activation associated with distinct cell backgrounds.

Similar to the BB loop, the DD loop in TLR5 likely extends beyond the TIR domain. For other TLRs, this loop has been shown to play a role in both homo- and hetero-dimerisation of TLRs and also between TLRs and adapters^25^; it is considered to play an important role in TIR-TIR interactions^21^. The DD loop region appears to have distinct characteristics only found in IL-1R, TLR5 and MyD88, namely a PI3K motif^17^. There is some evidence for PI3K activation being important for TLR5 signalling in humans and mice leading to enhanced^17,26^ or inhibited responses^27^ which may depend on distinct catalytic subunits of PI3K^28^. Our data provided evidence for a significant difference in PIK3 signalling by the bovine and human TLR5s in their cognate cell systems. CXCL8 production following bTLR5 activation required PI3K but this was not the case for hTLR5 in the HEK cells. Signalling differences may stem from variation in adaptor recruitment based on intracellular domains, such as the DD loop, of the respective TLRs. Our previous research has shown that there are amino acid differences between the TIR domain of bTL5 and hTLR5, including within the DD loop^2,7^. Our results differ to those of others^26^ that show PI3K is required for down-stream signalling by human TLR5. Ivison *et al*.^17^ suggested that the TLR5 DD loop contained a predicted PI3K tyrosine phosphorylation site at position 798–801: YQLM thus conforming to the PI3K motif YxxM. The authors showed that this tyrosine is phosphorylated following TLR5-flagellin binding, and mutation of the tyrosine at position 798 reduced signalling by human TLR5. Nevertheless, they did not show that hTLR5 recruits PI3K directly. Furthermore our data shows that while CXCL8 secretion in response to bTLR5 is PI3K-dependent (and hTLR5 not so), this PI3K motif is not present in bTLR5^7^ and yet it is able to signal. In fact, when we mutated the motif to instate the Tyrosine (bTLR5F798Y) this had a clear negative impact on the capacity of the bTLR to activate signalling in bovine cells, and a small but significant reduction of CXCL8 production in human cells at a lower concentration of flagellin.

However, our results indicate that PI3K activation is a necessary step in signalling from bTLR5-flagellin activation. It is unlikely that the motif region is phosphorylated by tyrosine kinases which then lead to PI3K recruitment. Instead this motif or local context within the DD loop may be important for effective signalling, potentially interacting with the BB loop of TLR5 itself during dimerization as well as the BB loop of MyD88 as indicated for other TLRs^13,15,21^. As suggested by Rhee *et al*.^26^, although in the context of hTLR5, TLR5-flagellin activation of PI3K is likely to be indirect with PI3K recruited via the adapter molecule MyD88. In fact several other TLR molecules are also phosphorylated by tyrosine kinases following ligand binding and some at least may also activate PI3K indirectly, although many details remain to be elucidated^29^. PI3K consists of a regulatory and catalytic subunit, with a number of different versions of both subunits, including splice variants, the expression of which lead to different effects in different cells^30^. The class IA *PIK3R1* gene encodes its regulatory subunit that consists of 3 splice variants, of which p85 is generally the most highly expressed. Knock-down of all three splice variants in HEK cells had no effect on hTLR5 activation, whereas in EBL siRNA specific for p85 significantly inhibited bTLR5 activation. Differences in the involvement of the class I catalytic subunits in TLR5 signalling in humans and mice have also been reported^28^.

The downstream pathways from TLR5/MyD88/PI3K involve TRAF6, NFκB and MAPKs including p38 and we found that activation of all three were essential for signalling by both human and bovine TLR5, confirming earlier human and mouse studies^22,27^. However, there were some differences in the way bTLR5 and hTLR5 signalling were affected by the siRNA duplexes and established inhibitors for the NFκB and p38 pathways. Knock down of RELA by siRNA inhibited the flagellin response by both hTLR5 and bTLR5. In agreement the NFκB inhibitor PDTC inhibited the response by hTLR5. In contrast, a greatly enhanced response was observed for bTLR5. RELA is the most common component of the NFκB complex, which can consist of distinct homo or hetero-complexes of at least five components. Thus, it is clear that both TLR5 molecules can act through NFκB, but there may be species differences in the other NFκB components involved that are differentially influenced by PDTC. The presence of the p38 inhibitor SB203580 resulted in inhibition of signalling by TLR5 of both species and knock down by siRNA for bovine p38 also prevented both molecules from signalling. However, the human p38 siRNA was a proprietary siRNA which successfully reduced expression of p38 transcripts, but did not inhibit the CXCL8 response by hTLR5. As p38 also consists of four distinct isoforms, it is possible that further subtle differences in the signalling pathways between humans and cattle TLR5 may be found. Additionally, inhibitors are not always completely specific for their main target molecule e.g. SB203580 has been reported to inhibit protein kinas B (or Akt)^31^ and possibly this may account for the lack of effect of the p38 siRNA in the HEK cells.

Our work also clearly challenges the conclusions of Osvaldova *et al*.^9^ who had suggested that because bTLR5 does not have Tyr at position 798, with the equivalent bovine sequence being F-H-L-M, it cannot stimulate a down-stream response to flagellin. They reported that exchange of the bovine F-H with a human Y-Q partially restored bovine bTLR5 activity in a HEK cell line, though not to the level seen with hTLR5. Our own data indicates that bTLR5 in the presence of flagellin induces a strong downstream response in various cell types including HEK cells^7^ and in fact the bTLR5F798Y variant in the bovine cell was unable to signal effectively. As we have shown in this paper, these discrepancies may be because bTLR5 does not interact optimally with human downstream adaptors and/or signalling molecules. The differences may also relate to the enhanced expression of bTLR5 in our system where over 90% of the cells were expressing bTLR5 at high levels. It is unclear what effects, if any, might be observed in our system if Q799H was also instated in bTLR5.

These studies confirm that we are only beginning to understand the complexity of the regulation underlying these critical and overlapping signalling networks. They emphasise that drawing universal conclusions from individual species can be over simplistic, and investigations between species in their responses should be considered in species relevant cells.

In addition to variation in signalling responses, it is possible that TLR5 has evolved in different host species to recognise distinct bacterial flagellins potentially reflecting the relative exposure of that host to that group of organisms and their pathogenic potential. Our earlier paper^2^ had revealed differences in the amino acid sequences between the ectodomains of human and bovine TLR5, some of which appear to be under selective evolutionary pressure. Since that paper was published only a soluble region of the extracellular domain of the fish species *Danio rerio* TLR5 has been co-crystallised with flagellin ligands, enabling flagellin binding sites to be identified^10,32^. In addition, an electron microscopy study has allowed a model to be built of human TLR5 dimers, with and without ligands^33^. Yoon *et al*.^10^ identified an important interaction of LRR9, which forms a flexible loop extending from the horseshoe conformation of TLR5, with the D1 domain of flagellin. A conserved residue, R90, in the flagellin D1 domain interacts with several amino acids in the LRR9 loop and plays an important role in the activation of human TLR5^11^, bovine TLR5^7^ and mouse TLR5^19^. Our previous work demonstrated a non-synonymous polymorphism in cattle as well as a ruminant specific site within the LRR9 region of bovine TLR5)^2^.

Thus, extracellular TLR5 variation may also result in host species-specific responses to distinct flagellins. TLR5 is currently only established to recognise the bacterial flagellin monomer as its activating ligand and while the two key regions of the flagellin D1 domain known to interact with TLR5 are relatively conserved, these regions can still vary substantially between bacterial species. While the *Salmonella* and *Escherichia* flagellin used in this study have very similar TLR5-interacting D1 sequences, these differ significantly in the *Listeria* and *Burkholderia* flagellins selected for our analysis. However, several amino acids are conserved between all five flagellin sequences. In particular, R90, E114 and Q97 make nearly 20% of the total interactions with the TLR5 binding sites. These form interactions with mainly conserved TLR5 contacts and may encompass a critical scaffolding for effective flagellin-TLR5 binding and signalling. Indeed Ala mutations in R90 or E114 have the greatest impact on zebra-fish TLR5 signalling^32^.

There are a few exceptions: R90 forms an H-bond with the base of the LRR9 cleft which is S267 in zebra-fish TLR5 and Ser is present at this site in most other species. However, primates, including humans express Ala at this site, and a few other species such as mice express Pro. The latter is sufficient to account for differences in flagellin interaction between humans and mice^34^.

We have also shown that additional positively selected sites in *Bos* and *Ruminantia* species exist, some of which (G104, S128, Q207 and G239) interact with conserved flagellin sites (Q89, Q97 and A427).

A further three *Bos*-specific exchanges (F179, K181 and R261) might account for differences between bTLR5 and hTLR5 in their activation by *B. thailandensis* and *L. ivanovii* flagellins, as these three sites interact with distinct binding amino acids in these flagellins (*L. ivanovii*: D78, S82, S419, R433; *B. thailandensis*: S78, T82, N419, Q422). Although our data is preliminary, flagellin from *B. thailandensis* activated hTLR5 to a greater degree than *L. ivanovii* flagellin whereas the reverse was observed for bTLR5. Nonetheless, the flagellins were only compared at a single dose and their binding kinetics could provide data that are more conclusive. Further mutagenesis experiments are also warranted to test whether these differences in host and flagellin sequences may result from different host-pathogen life histories. *Burkholderia* flagellin has an identical sequence to that expressed by the important human pathogen *Burkholderia pseudomallei*, which can also cause disease in many animals including cattle^35^ whereas *L. ivanovii* is a ruminant specific pathogen^36^.

In conclusion we have confirmed that bTLR5 is fully functional, and that both bTLR5 and hTLR5 signal more effectively following flagellin interaction when they are expressed in cells containing cognate signalling partners. Comparison of the TIR sequences of TLR5 would suggest that the most likely candidate is the initial adapter, MyD88, but other signalling partners cannot be ruled out, as we found subtle differences in responses in the presence of specific inhibitors and siRNA knock-down of known components of TLR signalling. Sequence comparisons of different flagellins which have distinct host outcomes may interact with distinct host TLR5 binding sites and analysis of these differences has suggested further targets for mutation and confirmation of structure-function relationships. Species differences may also impact on the use of flagellin as a vaccine adjuvant. Thus delineating species specific activation and signalling is required for the most effective exploitation of this activity and to better understand host-bacterium co-evolution in different animal hosts.

## Materials and Methods

### Flagella expression and purification

*E. coli* H7 (Ec H7), *S*. Typhimurium P1 (St P1), *Burkholderia thailandensis* FliC (Bt FliC) and *Listeria ivanovii* PAM55 FlaA (Li FlaA) flagella were prepared and purified as described previously for *E. coli* ^7,37^. *Listeria ivanovii* PAM 55^36^ was cultured overnight in 100 ml brain-heart infusion (BHI, Difco-BD) and then diluted 1:100 into fresh BHI and cultured at 22 °C with shaking [200 rpm] until stationary phase. Bacteria were harvested by centrifugation [4,500 rpm, 4 °C, 7 min]. *B. thailandensis* ^38^ was cultured in LB broth for flagella extraction following the same methodology.

All flagella preparations were stored at −20 °C in PBS. All flagella underwent rigorous lipopolysaccharide (LPS) removal as described previously^7^. In brief this was repeated extraction in 1% Triton × 114, with the final aqueous phase for each species’ flagella applied to Pierce® High-Capacity Endotoxin binding resin following the manufacturer’s instructions (Thermo Scientific). Endotoxin levels were assessed as described previously^7^. Before use in experiments all flagella were monomerised by heat treatment^7^. Flagella were then stored in aliquots at −20 °C.

### Cell lines

In our previous work^7^, Human Embryonic Kidney (HEK) cells were used, but we observed that un-transfected HEK cells did have a low level response to flagellin, potentially because of some endogenous expression of human TLR5. In this study we switched to using HEK-293T cells, as in our hands these un-transfected cells showed no CXCL8 production in response to flagellin at any concentration (see Fig. 1) in contrast to HEK cells^7^. Un-transfected EBL also did not respond to increasing levels of flagellin, although they did express low levels of CXCL8 (See Fig. 1). HEK-293 T (HEK) cells and embryonic bovine lung epithelial cells (EBL) (Leibniz Institute DSMZ-German Collection of Microorganisms and Cell Cultures) were cultured at 5% CO_2_ and 80% humidity in Dulbecco’s modified Eagle’s medium (DMEM) supplemented with 10% (v/v) FBS (Sigma-Aldrich, UK), 1 U of penicillin (Invitrogen Life Technologies, BV, Netherlands), 1 µg/mL of streptomycin (Invitrogen) and 2 mM L-glutamine (Invitrogen).

### TLR5 clones

The full length of bovine TLR5 was amplified and sub-cloned into the modified ptGFP1 expression vector as previously described^7,12^. A similar approach was carried out to amplify hTLR5 (InvivoGen, Toulouse, France) using specific primers encoding the full length of the gene alongside restriction enzymes sites HindIII and SacII: hTLR5ptGFP-FW:CTGAGCAAGCTTCACCATGGGAGACCACCTGGAC, hTLR5ptGFP-RV: CGACATCCGCGGTTAGGAGATGGTTGCTAC.

As previously described, amplified products were transformed into competent XL1-Blue Competent Cells (Stratagene, Agilent Technologies Division, USA); plasmid DNA from four independent colonies was purified and sent for sequencing to confirm correct sequences. The confirmed hTLR5 plasmid and the modified ptGFP1 vector were then digested with HindIII and SacII, correct fragments purified, and ligated overnight and transformed into competent JM109 cells (Promega, Madison, USA), with selection on LB agar with kanamycin (50 µg/ml), followed by culturing overnight in LB medium with kanamycin (50 µg/ml). Plasmid DNA was purified using a QIAprep Plasmid DNA Miniprep kit (Qiagen Inc., Netherlands) and concentrations determined by spectrophotometry (Nanodrop, Labtech International, UK).

### Site-directed TLR5 mutagenesis

The site directed mutation, F798Y in the cytoplasmic domain of TLR5 was generated using the QuikChange Site-Directed Mutagenesis Kit (Agilent technologies), following the manufacturer’s protocols. The primers used for these reactions were: forward primer 5′-GTCCCTGTCCCAGT**A**CCATCTGATGAGGC-3′ and reverse primer 5′-GCCTCATCAGATGGTACTGGGACAGGGAC-3′. Amplified and treated constructs were transformed into *E. coli* XL10-Gold Ultra competent bacteria and plated on *E. coli* Fast-Media® Blas agar plates (InvivGgen) and incubated overnight on 37 °C. Plasmids were extracted, sequenced (DNA Sequencing by GATC Biotech, Germany) and analysed using Lazergene software (http://www.dnastar.com). The mutated F798Y bTLR5 was then sub-cloned into the modified ptGFP1 vector and F798Y bTLR5 expressing cell lines derived according to the method described below.

### Transfection of cell lines with TLR5 clones

For transfection, EBL and HEK-293T cells were incubated in supplemented DMEM as described above. Cells were passaged 48 h prior to transfection by electroporation with 2 μg of bTLR5-ptGFP1, hTLR5-ptGFP1 construct or empty ptGFP1 vector (control) in a Nucleofector™ 2d Device (Lonza Group Ltd., US) as detailed previously^7^. After 24 h, all media were replaced with fresh media supplemented with 500 μg/ml of neomycin (G418, Sigma Aldrich). This was replenished every day to remove dead cells, and the concentration of neomycin reduced to 250 μg/ml, one week following transfection.

Once around 25–50% of the cells were GFP positive, they were cell sorted using a FACSAria™ III which generated cultures of >90% GFP^+^ cells after one or more rounds of sorting, except for the F798Y mutant in EBL which persistently through several rounds of sorting contained a small proportion of GFP^-^ cells (Supplementary Fig. S1). Once the desired enrichment was achieved each cell line was frozen in aliquots. RT-PCR was used to confirm the expression of the constructs in the transfected EBL and HEK-293T cell line, including empty vector control lines. The primers used for identification of bovine TLR5 and endogenous control bovine GAPDH (bGAPDH) in the bTLR5-ptGFP1 and F798Y-bTLR5-ptGFP1 cell lines are detailed elsewhere^7^. The forward primer for hTLR5 matched sequence in the 3′ end of human TLR5 (hTLR5-FW: AGAAAACCGCATTGCCAATATC) whilst the reverse primer (ptGFP1-hTLR5-RV: CTGATTATGATCTAGAGCCGCGGTTAGGAGA) was designed to span both hTLR5 and ptGFP1. The reactions (25 μl) were an initial denaturation of 2 min at 95 °C, followed by 35 cycles of 95 °C for 30 s, 60 °C for 30 s and 72 °C for 45 s. All PCR products were visualised on a 1% agarose gel containing SYBR® Safe DNA Gel Stain (Life Technologies, BV, Netherlands). Each construct was present in both EBL and HEK cell lines (Supplementary Figs S7 and S8).

### CXCL8 ELISA

The read-out for TLR5 activity in all experiments was CXCL8 secretion into cell line culture supernatants. Cell lines were prepared at 5 × 10^5^ cell per ml and 180 μl/well was added to each well in a 96-well plate, with the addition of 20 μl/well flagellin at final concentrations as required (see results). Following overnight incubation, cell culture supernatants were harvested for assays. Human CXCL8 protein levels were measured by ELISA using the BD OptEIA human IL-8 ELISA set (BD Biosciences) following the manufacturer’s instructions. Bovine CXCL8 protein release was measured by ELISA as described previously^7^. All ELISAs were carried out using Nunc-Immuno Maxisorp 96 well plates, with all samples and standards in triplicate. The plates were read using a Synergy HT plate reader (BioTek).

### Inhibitors of innate signalling pathways

Cell lines were prepared at 5 × 10^5^ cell per ml and the following inhibitors were added: PI3K inhibitor LY294002 (50 μM), (Calbiochem, San Diego, CA, USA), p38α and p38β inhibitor, SB203580 (5 μM) (InvivoGen) and NFκB inhibitor, PDTC (100 μM) (AbMole BioScience Inc., Brussels, Belgium), with DMSO as a vehicle control. The cells were vortexed and 180 μl/well of the cells were added to 96 well plates. The cells were incubated for an hour before adding H7 flagellin at a final concentration of 25ng/ml. The cell lines were then incubated overnight, and supernatants were collected for CXCL8 ELISA analysis. Each experiment was carried out in triplicate and repeated three times.

### siRNA knock-down

Previously designed and validated siRNA for human p38, RELA, TRAF6 and PIK3R1 were obtained from Sigma-Aldrich (Supplementary Table S2). Three siRNA duplexes for the bovine homologue of each gene were designed by Sigma-Aldrich and assessed for their ability to knock-down target gene mRNA levels. One siRNA for each gene, which gave optimal knock-down with minimum off-target effects, was chosen for the analysis (Supplementary Table S3), except for PIK3R1. In addition, the AllStars negative control siRNA (Qiagen), which does not share homology with any known mammalian gene, was used as a non-target siRNA control (NTC).

For the siRNA experiments HEK-hTLR5 and EBL-bTLR5 cell cultures were prepared at 2 × 10^5^ and 1 × 10^5^ cells per ml respectively and 1 ml/well was added to each well of a 12 well plate. After 24 hr HEK-hTLR5 cells were transfected with siRNA following the manufacturer’s protocol at predetermined optimal conditions, initially generating a mix of 5 μl Lipofectamine RNAiMAX and 5 μl 20 μM siRNA in 200 μl Opti-MEM I reduced serum medium (Invitrogen). After 20 min incubation at room temperature the siRNA/Lipofectamine RNAiMAX mix was added to the cells in 1 ml medium, giving a final concentration of 83.3 nM siRNA. EBL-bTLR5 cells were transfected in a similar manner, using 4 μl Lipofectamine RNAiMAX and 4 μl 20 μM siRNA in 200 μl Opti-MEM I reduced serum medium (Invitrogen), giving a final concentration of 66.7 nM siRNA. Additional controls included in each experiment were cells treated with Lipofectamine RNAiMAX only (transfection control, TC) and untreated cells (negative control, NC). After 24 hr the cells were stimulated with 100 ng/ml *E. coli* H7 flagellin. Cells were harvested at 4 hr post stimulation for RNA extraction and supernatants were collected 24 hr post stimulation for CXCL8 ELISA analysis. Each experiment was carried out in triplicate and repeated three times.

### Reverse transcription: quantitative PCR (RT-qPCR) analysis of target gene knock-down

Total RNA was extracted from all HEK-hTLR5 and EBL-bTLR5 samples using the ReliaPrep RNA extraction kit (Promega), including a DNase digestion step, according to the manufacturer’s instructions. First strand cDNA was reverse transcribed from 0.5 μg total RNA using an oligo(dT) primer and GoScript reverse transcriptase (Promega) according to the manufacturer’s protocols. The resulting cDNA was diluted 1:20 for RT-qPCR analysis. Pre-designed oligonucleotides for human p38, RELA, TRAF6 and CHAMP1 were used in the analysis (Sigma-Aldrich) (Supplementary Table S3). Oligonucleotides for human PIK3R1 and bovine target genes were designed using Primer3^39^ and Netprimer (Biosoft International) software (Supplementary Table S3). The mRNA level of each transcript was quantified by qPCR as described previously^7^. CHAMP1 was found to be a suitable reference gene, with stable expression across HEK and EBL samples (data not shown), and was used to standardize the results for the genes of interest. All siRNAs led to specific knock-down of mRNA, with no off-target effects except for PI3KR#b1 (results not shown) which was excluded in the final analysis (Supplementary Fig. S9 and see Results section). The average level of knock-down of the four target genes in HEK-hTLR5 cells ranged from 58.3% for PIK3R1 to 69.5% for RELA (Supplementary Fig. S9(a)). A higher level of knock-down was achieved in EBL-bTLR5 cells. The average level of knock-down ranged from 79.2% for p38 to 89.1% for PIK3R1 with PIK3R1#b3 siRNA (Supplementary Fig. S9(b)).

### Sequence alignment and comparative analysis of TLR5 and flagellin domains

Nucleotide sequences encoding TLR5 were obtained from GenBank and Ensembl and the horse TLR5 sequence was kindly provided by Dr James MacLeod, University of Kentucky, USA (Supplementary Table S1). Translated protein sequences were aligned using Clustal Omega^40^ and visualised using Jalview^41^. Domains and putative functional sites were mapped according to^2^ and^10^ for TLR5 and by analogy with other TIR domain proteins^13,14^. The following flagellin sequences were obtained from Uniprot, their Nt and Ct D1 domains aligned and amino acids involved in TLR5 binding mapped according to published data^10,11^: *B. thailandensis* FliC (WP_009906598.1), *E. coli* 0157 H7 (Q7AD06), *L. ivanovii* (strain ATCC BAA-678 / PAM 55) FlaA, (G2ZCV3), *S*. Typhimurium str. FliC (P06179) and *S*. Dublin FliC (Q06971).

### Statistical analysis

All experiments were conducted on three separate occasions using separate aliquots of cell lines and flagella, and with triplicate technical replicates. All statistical analyses were carried out using Minitab version 17. All the data were log transformed before statistical analysis which accounted for both the within and between experiment variance. The effects of siRNA and inhibitors were analysed using an ANOVA model fitting experiment as random effects and treatment (siRNA or inhibitor) as fixed effects. Subsequent Tukey’s tests were used to identify significant differences between treatments.

### Data Availability

All data generated or analysed during this study are included in this published article (and its Supplementary Information files) and TLR5 sequences used in the alignments were obtained from Genbank and/or Uniprot (See Supplementary Table S1).

## Electronic supplementary material

Supplementary Information
