# Supplementary material for: Host species adaptation of TLR5 signalling and flagellin recognition
Source: Sci Rep. 2017 Dec 15;7:17677. doi: 10.1038/s41598-017-17935-5 (PMC5732158; doi:10.1038/s41598-017-17935-5)
Supplement: Supplementary file 1 — Supplementary Information [file 41598_2017_17935_MOESM1_ESM.doc]

**Supplementary Information**

**Host species adaptation of TLR5 signalling and flagellin recognition**

Amin Tahoun^#1,2^, Kirsty Jensen^#1^, Yolanda Corripio-Miyar^1,3^, Sean McAteer^1^, David G.E. Smith^4,5,6^ , Tom N. McNeilly^4^, David L. Gally^1^, Elizabeth J. Glass^1*^

^1^Division of Immunity and Infection, The Roslin Institute and R(D)SVS, The University of Edinburgh, Easter Bush, Midlothian, EH25 9RG, UK

^2^Present address: Faculty of Veterinary Medicine, Kafrelsheikh University, 33516 Kafr el-Sheikh, Egypt

^3^Present address: Moredun Research Institute, Pentlands Science Park, Bush Loan, Penicuik, EH26 OPZ, UK

^4^Moredun Research Institute, Pentlands Science Park, Bush Loan, Penicuik, EH26 OPZ, UK

^5^University of Glasgow, Glasgow G12 8TA, UK.

^6^Present address: Institute of Biological Chemistry, Biophysics and Bioengineering, Heriot Watt University, Edinburgh, EH14 1AS.

^#^These authors contributed equally to the work.

*Corresponding author: Prof. Liz Glass, The Roslin Institute and Royal (Dick) School of Veterinary Studies, University of Edinburgh, Edinburgh EH25 9RG, UK.

Telephone: +44 (0)131 6519242

email: [Liz.Glass@roslin.ed.ac.uk](mailto:Liz.Glass@roslin.ed.ac.uk)

Author email addresses:

[amin12_veta@yahoo.com](mailto:amin12_veta@yahoo.com)

[kirsty.jensen@roslin.ed.ac.uk](mailto:kirsty.jensen@roslin.ed.ac.uk)

[Yolanda.Corripio-Miyar@moredun.ac.uk](mailto:Yolanda.Corripio-Miyar@moredun.ac.uk)

[sean.mcateer@roslin.ed.ac.uk](mailto:sean.mcateer@roslin.ed.ac.uk)

[david.smith@hw.ac.uk](mailto:david.smith@hw.ac.uk)

[Tom.McNeilly@moredun.ac.uk](mailto:Tom.McNeilly@moredun.ac.uk)

[dgally@ed.ac.uk](mailto:dgally@ed.ac.uk)

[Liz.Glass@roslin.ed.ac.uk](mailto:Liz.Glass@roslin.ed.ac.uk)

Supplementary Figures S1-9 and Supplementary Tables S1-S3

**Supplementary Figure S1 Expression of transfected constructs b, h or mutant F798Y bTLR5-ptGFP1 in freshly cultured EBL and HEK293T as determined by eGFP positivity in flow cytometry.**


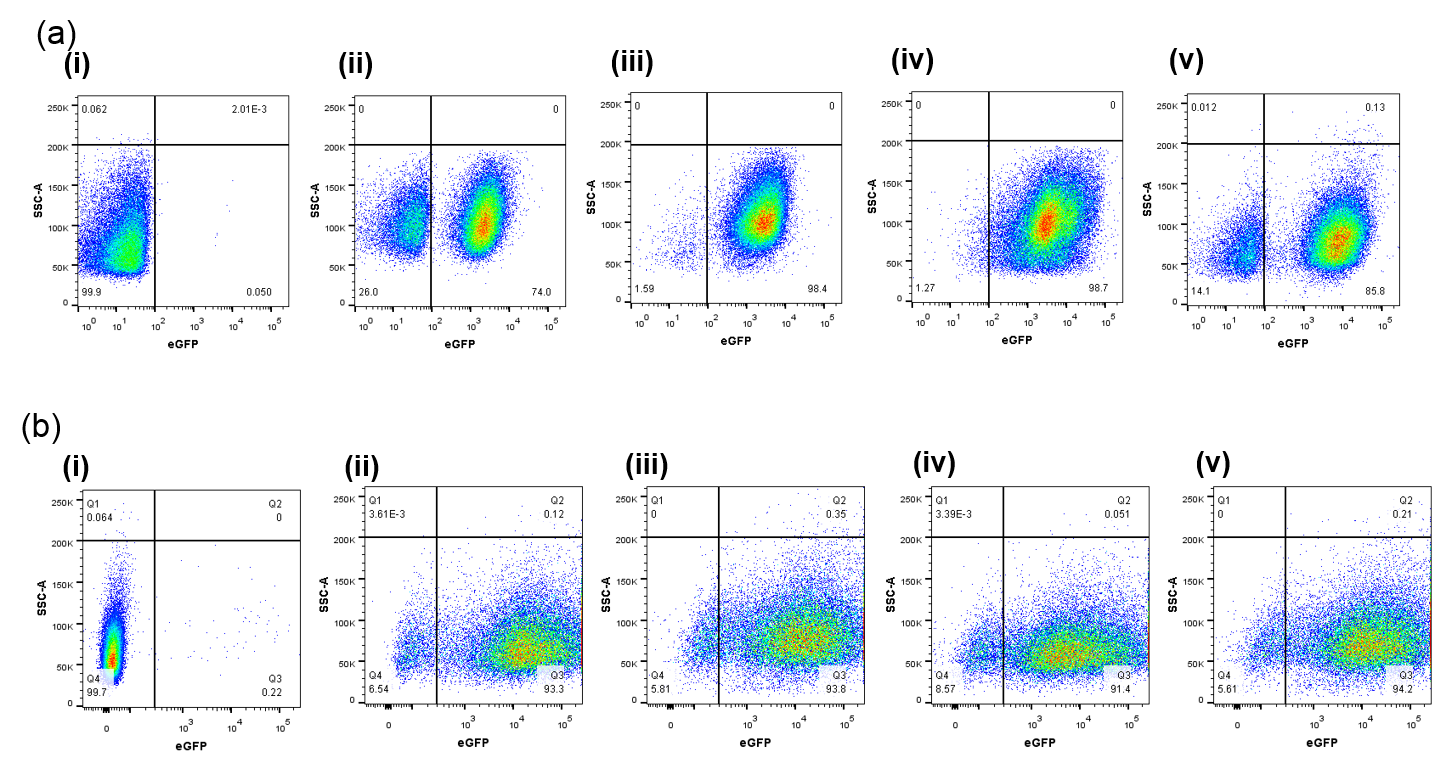


(a) The percentage of GFP positive cells in EBL: non-transfected EBL (i); transfected with empty vector (ii); transfected with bTLR5-ptGFP1 (iii); transfected with hTLR5-ptGFP1 (iv); transfected with F798Y mutant bTLR5-ptGFP1 TLR5-ptGFP1 (v).

(b) The percentage of GFP positive cells in HEK293T: non-transfected HEK293T cells (i); transfected with empty vector (ii); transfected with bTLR5-ptGFP1 (iii); transfected with hTLR5-ptGFP1 (iv); with F798Y mutant bTLR5-ptGFP1 (v)

All cells were gated to eliminate dead cells and doublets and live, single cells were further analysed for the expression of eGFP.

**Supplementary Figure S2 The activity of bovine and human TLR5 differs with cell background.**

**
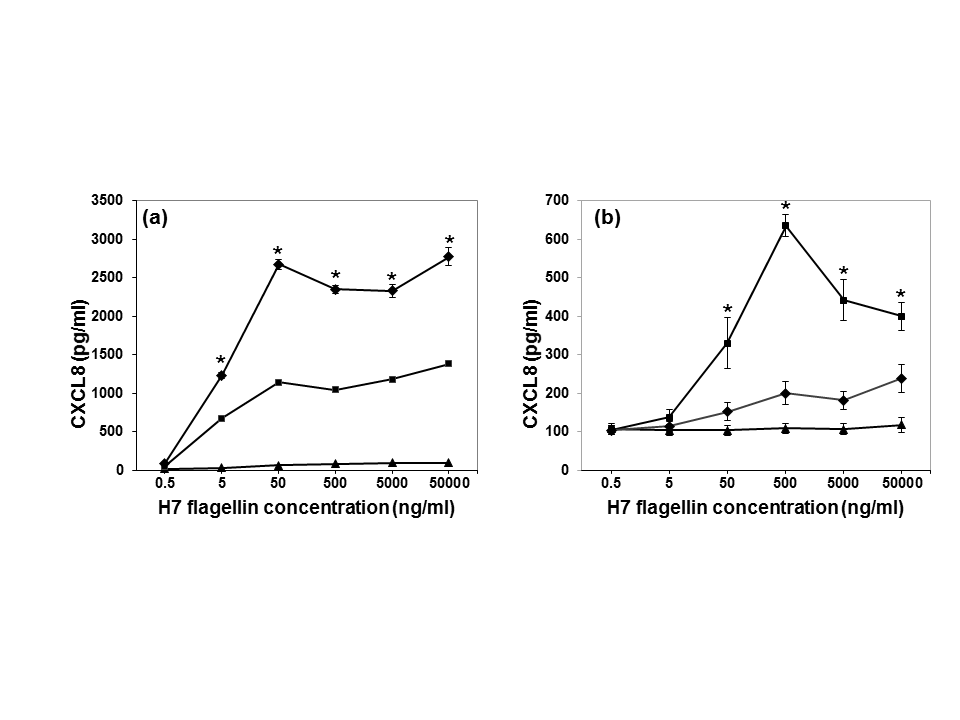
**

CXCL8 protein release from (a) HEK293T cells and (b) EBL cells stably transfected with human TLR5 (♦), bovine TLR5 (■) and untransfected cells (▲), activated with *E. coli* H7 flagellin at various concentrations. The Figures show the average CXCL8 protein concentration in pg/ml for each H7 concentration and the error bars illustrate the standard error of the mean for three experiments, each with three technical replicates. The average CXCL8 protein release in response to flagellin in pg/ml was significantly different in cells expressing bovine or human forms of TLR5 by ANOVA (P<0.001) and * denotes which doses were significant by subsequent Tukey’s test (P<0.05).


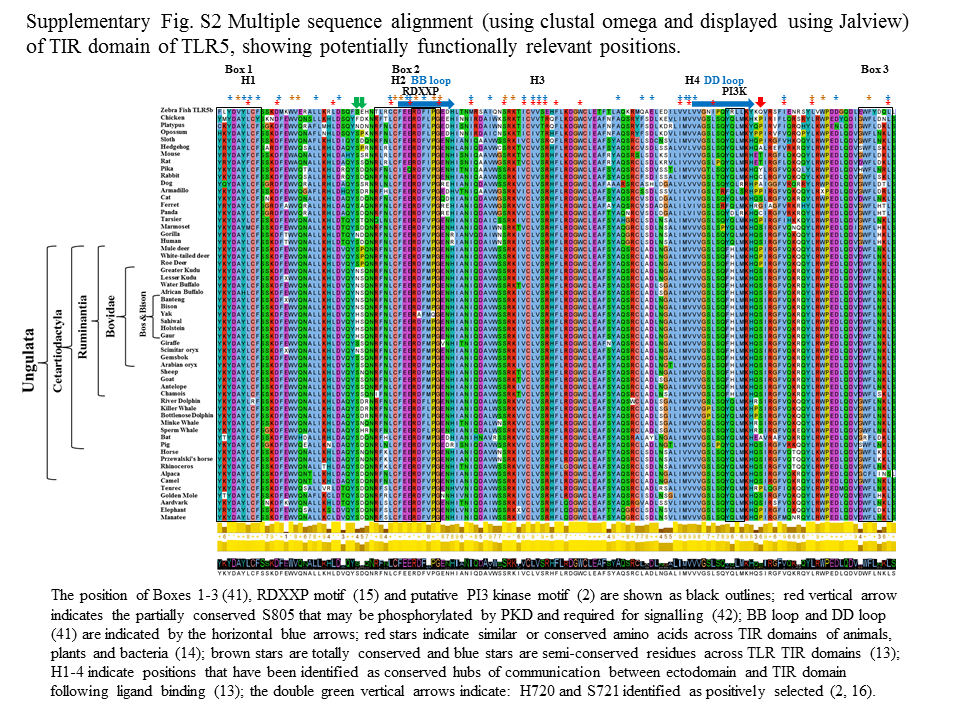
**Supplementary Figure S3 Multiple sequence alignment of TIR domain of TLR5.**

Potentially functionally relevant positions are shown using clustal omega and displayed using Jalview. The position of Boxes 1-3 (41), RDXXP motif (15) and putative PI3 kinase motif (2) are shown as black outlines; red vertical arrow indicates the partially conserved S805 that may be phosphorylated by PKD and required for signalling (42); BB loop and DD loop (41) are indicated by the horizontal blue arrows; red stars indicate similar or conserved amino acids across TIR domains of animals, plants and bacteria (14); brown stars are totally conserved and blue stars are semi-conserved residues across TLR TIR domains (13); H1-4 indicate positions that have been identified as conserved hubs of communication between ectodomain and TIR domain following ligand binding (13); the double green vertical arrows indicate: H720 and S721 identified as positively selected (2, 16).

**Supplementary Figure S4 The effect of a mutation in the cytosolic region of bovine TLR5 differs with cell background.**

**
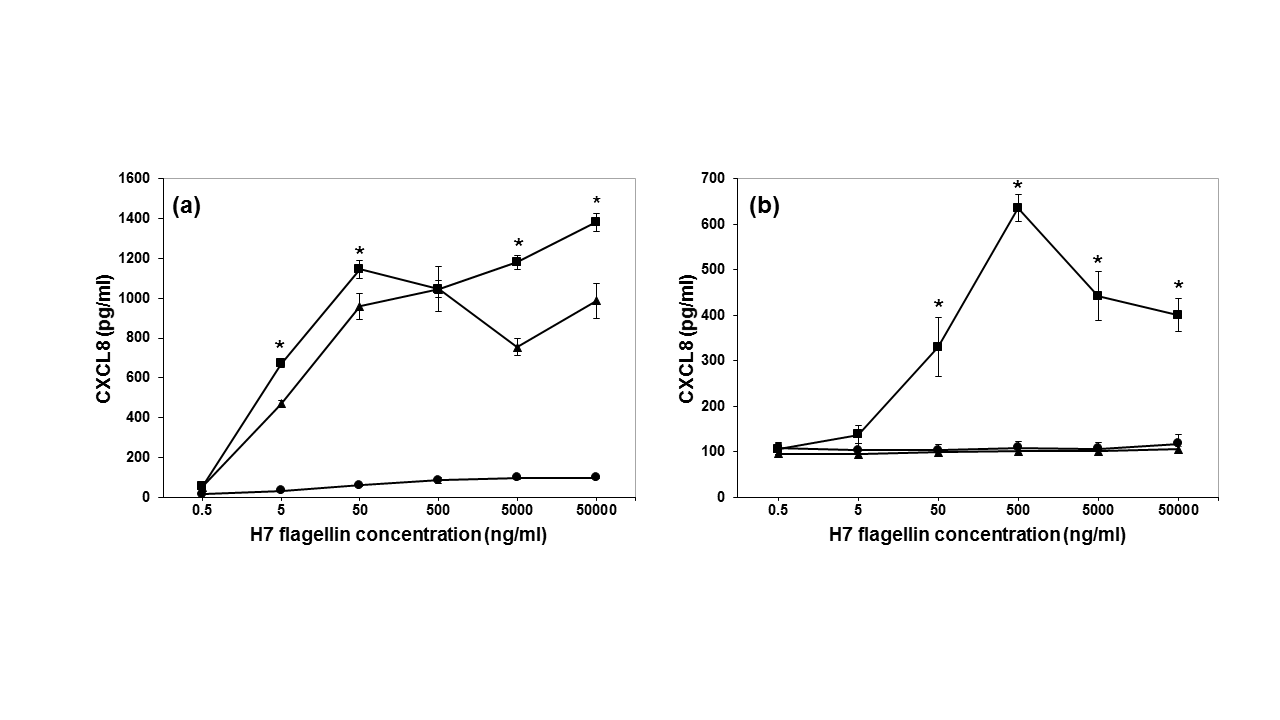
**

CXCL8 protein release from (a) HEK293T cells and (b) EBL cells stably transfected with bovine TLR5 wild-type TLR5 (■), bovine F798Y mutant TLR5 (▲) and untransfected cells (●), activated with *E. coli* H7 flagellin at various concentrations. The Figures show the average CXCL8 protein concentration in pg/ml for each H7 concentration and the error bars illustrate the standard error of the mean for three experiments, each with three technical replicates. The average CXCL8 protein release in response to flagellin in pg/ml was significantly different in cells expressing bovine wild-type or bovine F798Y forms of TLR5 by ANOVA (P<0.001) and * denotes which doses were significant by subsequent Tukey’s test (P<0.05).


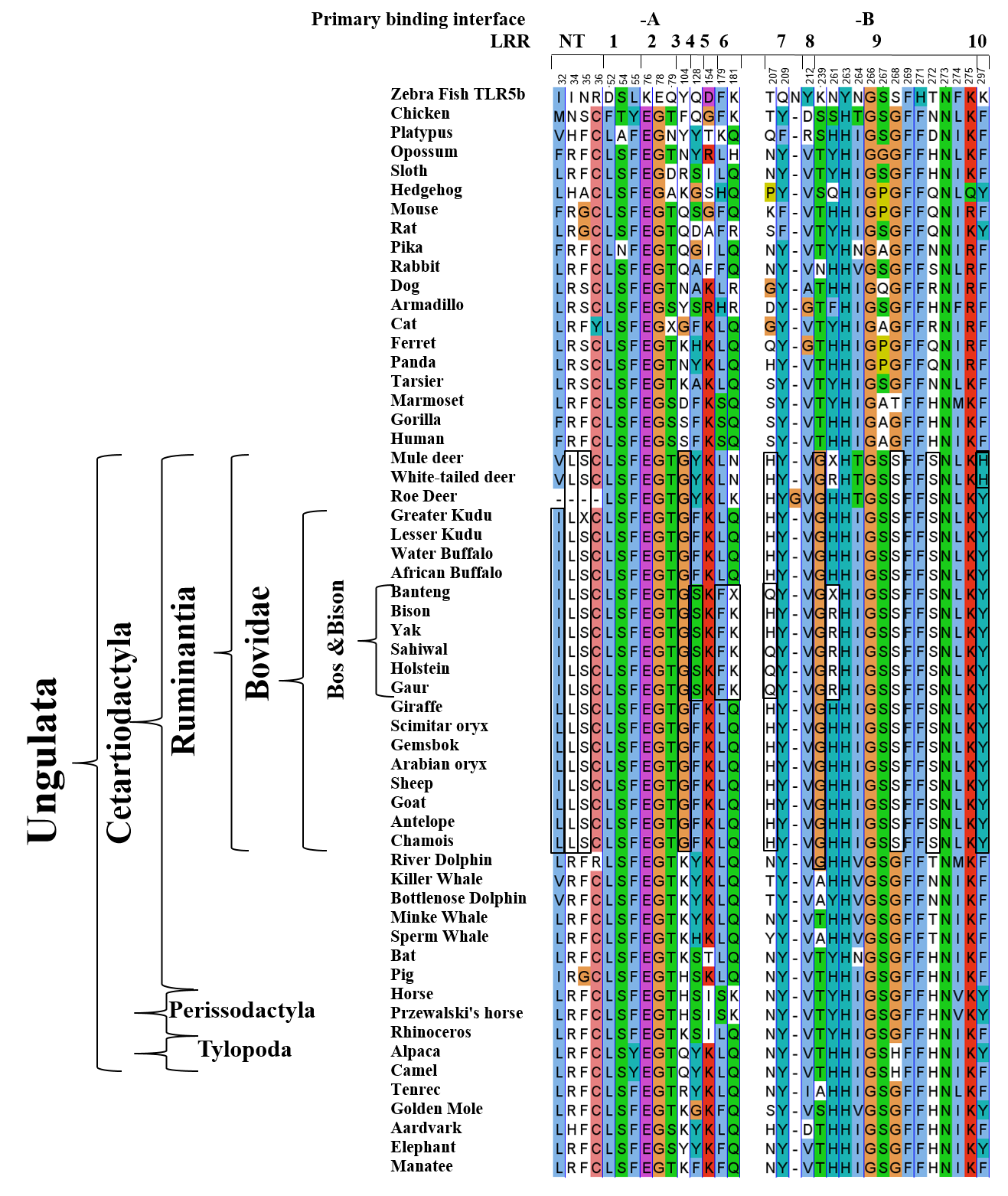
**Supplementary Figure S5 TLR5 Multiple sequence alignment showing only the ectodomain amino acids involved in binding flagellin.**

Flagellin binding amino acids were identified according to Yoon et al (10). Numbering of amino acids according to Smith et al (2) for bovine TLR5. Multiple alignment was conducted using clustal omega and displayed using Jalview. Black boxes highlight amino acids that are *Ruminantia* or *Bos* (and *Bison*) specific.


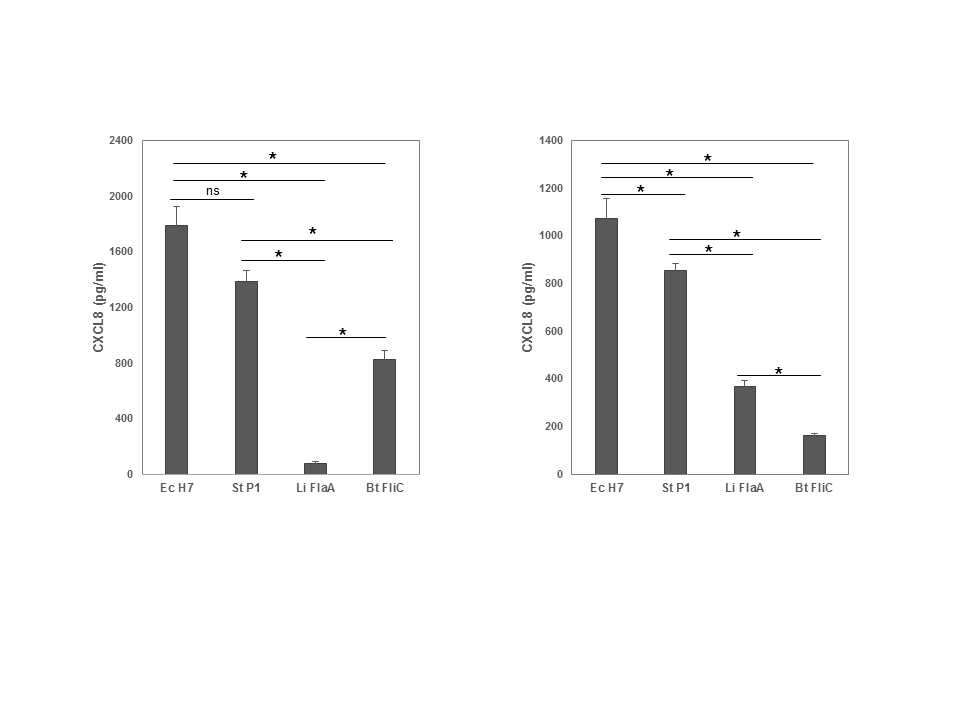
**Supplementary Figure S6** **The ability of bovine and human TLR5 to recognize flagellins from various bacteria differs.**

**(a) (b)**

The average CXCL8 protein release from (a) HEK293T cells stably transfected with human TLR5 and (b) EBL cells stably transfected with bovine TLR5 after stimulation with 50ng/ml *E. coli* H7 (Ec H7), *S.* Typhimurium P1 (St P1), *Listeria ivanovii* FlaA (Li FlaA) and *Burkholderia thailandensis* FliC (Bt FliC). Error bars illustrate the standard errors of the mean for three experiments, each with three technical replicates. * denotes that the average CXCL8 protein release in response to the different flagellin was significantly different by ANOVA and subsequent Tukey’s test (P<0.05). In this set of experiments 50ng/ml for Ec H7 was initially determined to be optimal.


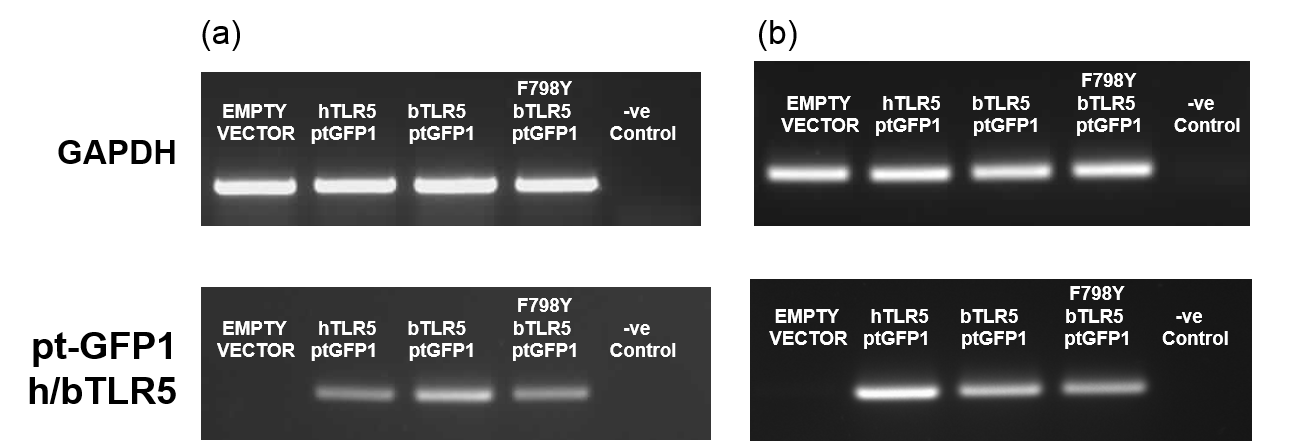
**Supplementary Figure S7 Expression of transfected constructs b, h or F798Yb TLR5-ptGFP1 in freshly cultured EBL and HEK293T as determined by RT-PCR.**

(a) Expression of constructs in EBL transfected with: empty ptGFP1 vector, hTLR5-ptGFP1, bTLR5-ptGFP1 or F798Y mutant bTLR5-ptGFP1 by RT-PCR. The bGAPDH primer set was used as an endogenous control for the cDNA and specific primers for ptGFP1 (empty vector), hTLR5, bTLR5 and mutant F798Y bTLR5 were used to determine the presence of each construct in the transfected cell lines.

(b) Expression of constructs in HEK293T transfected with: empty ptGFP1 vector, hTLR5-ptGFP1, bTLR5-ptGFP1 or F798Y-ptGFP1 by RT-PCR.

The hGAPDH primer set was used as an endogenous control for the cDNA and specific primers for ptGFP1 (empty vector), hTLR5, bTLR5 and mutant F798Y bTLR5 were used to determine the presence of each construct in the transfected cell lines.

Lanes on the gels are as indicated by the labels. (The gels shown are cropped and derived from 4 separate full-length gels which are shown in complete form in Supplementary Figure S8).

**Supplementary Figure S8 Expression of transfected constructs b, h or F798Yb TLR5-ptGFP1 in freshly cultured EBL and HEK293T as determined by RT-PCR**. Full length gel images of the cropped images shown in Supplementary Figure S4 with relevant lanes as indicated by the labels


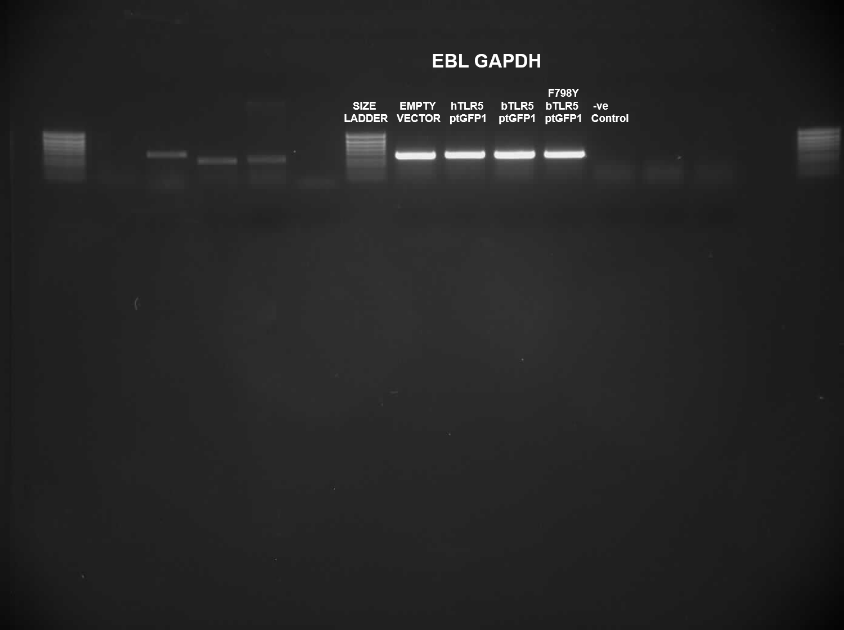

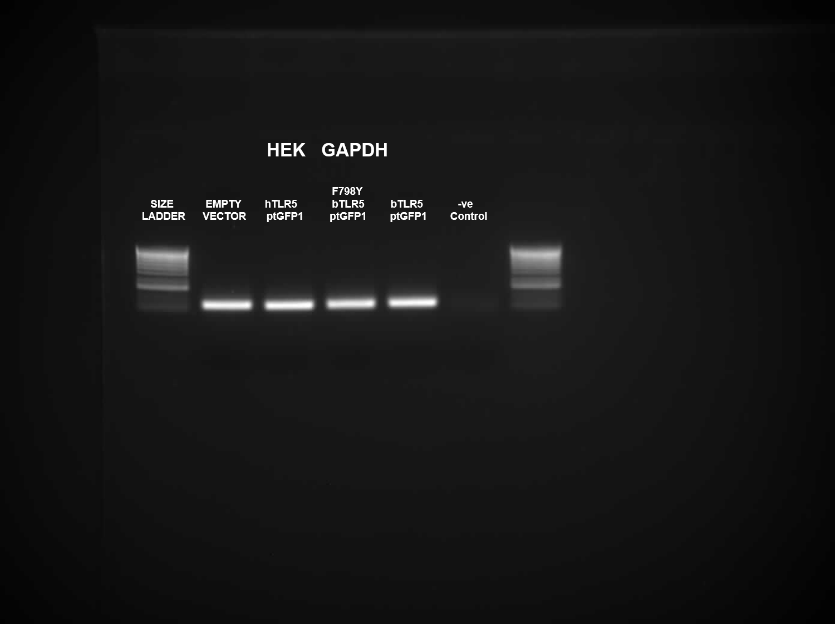

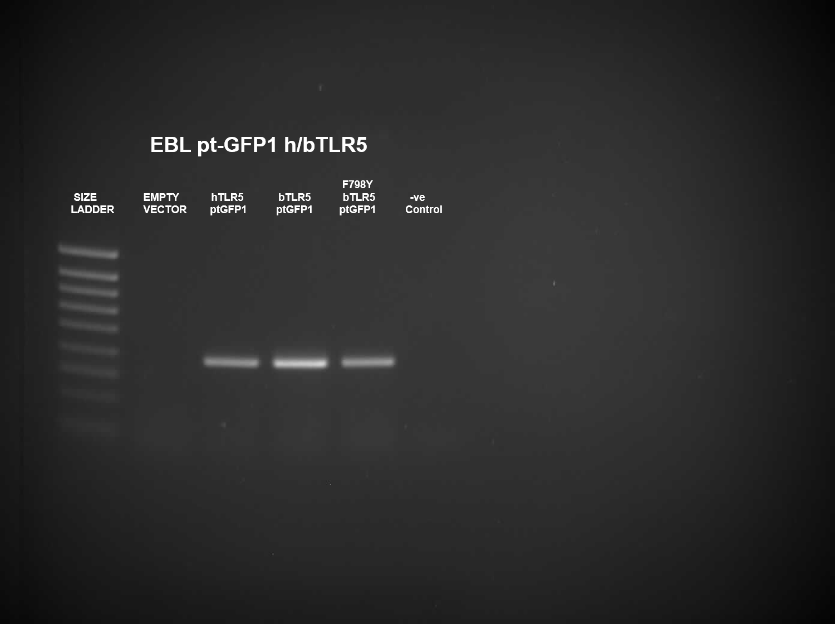

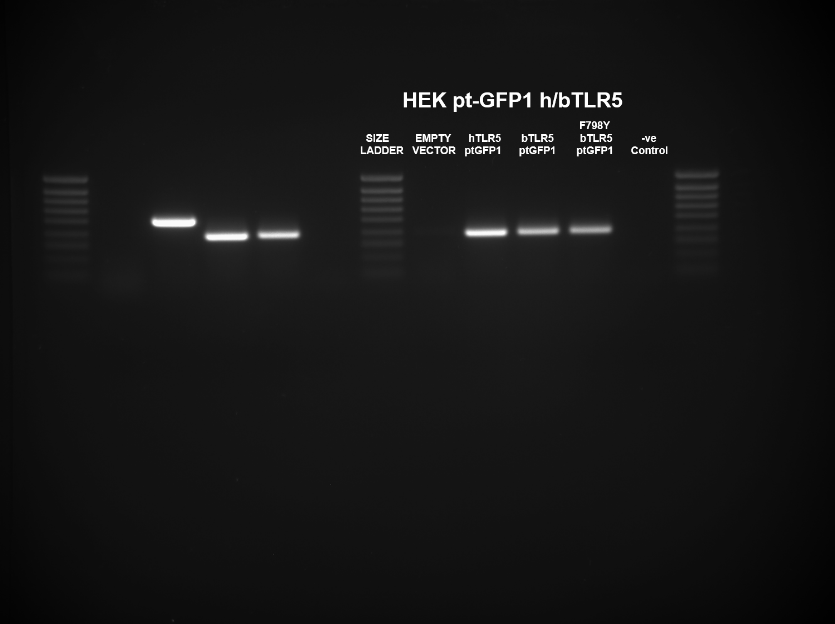


Figure Legend see Supplementary Figure S7.

**Supplementary Figure S9** Quantification of target gene knock-down in (a) HEK293T-hTLR5 cells and (b) EBL-bTLR5 cells.


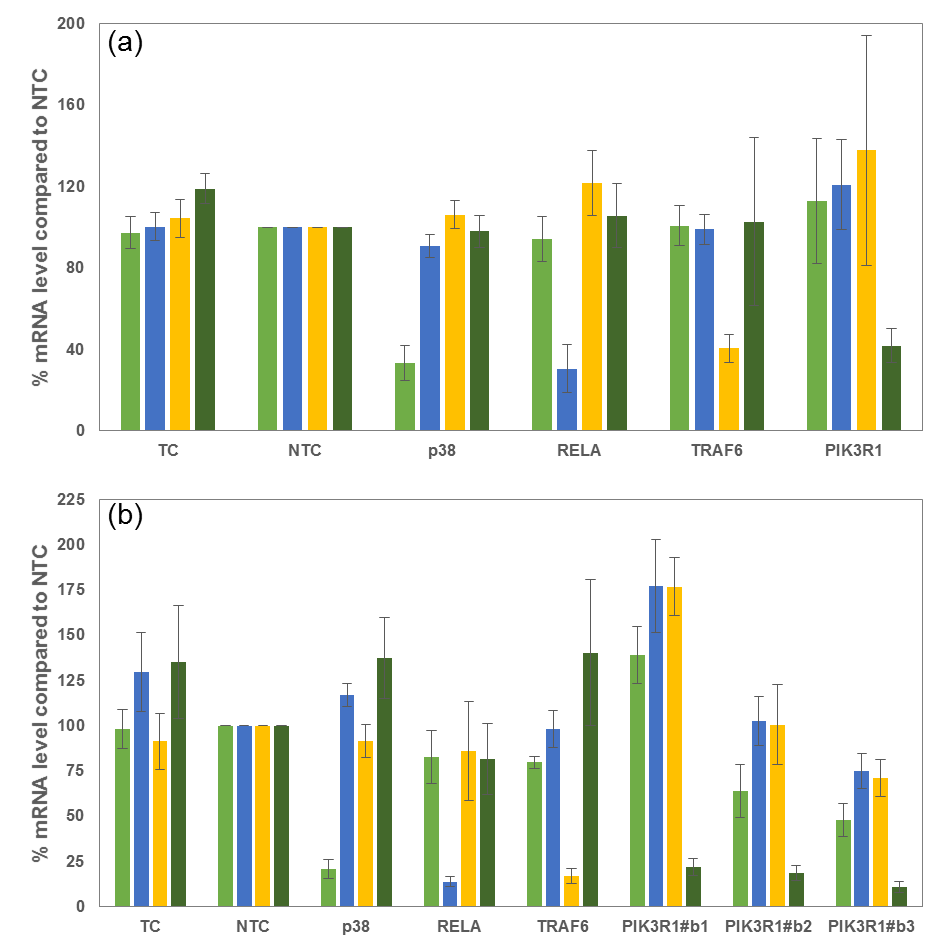


The graphs illustrate the average percentage mRNA levels of p38 (light green bars), RELA (blue bars), TRAF6 (yellow bars) and PIK3R1 (dark green bars) for samples treated with each target gene siRNA compared to samples treated with non-target control (NTC) siRNA. TC denotes cells treated with transfection reagent only. Error bars illustrate the standard deviation of triplicate repeats

Supplementary Table S1 List of TLR5 mRNA and protein accession numbers

| **Common Name** | **Species Name** | **mRNA** | **Protein ID** |
| --- | --- | --- | --- |
| Aardvark | *Orycteropus afer afer* | [XM_007936955.1](http://www.ncbi.nlm.nih.gov/nuccore/634837302) | [XP_007935146.1](http://www.ncbi.nlm.nih.gov/protein/634837303) |
| African forest buffalo | *Syncerus caffer nanus* | [JN615234.1](https://www.ncbi.nlm.nih.gov/nuccore/373159247) | [AEY63775.1](http://www.ncbi.nlm.nih.gov/protein/373159248) |
| Alpaca | *Vicugna pacos* | [XM_006220179.2](http://www.ncbi.nlm.nih.gov/nuccore/970704835) | [XP_006220241.2](http://www.ncbi.nlm.nih.gov/protein/970704836) |
| American bison | *Bison bison* | [JN615236.1](http://www.ncbi.nlm.nih.gov/nuccore/373159251) | [AEY63777.1](http://www.ncbi.nlm.nih.gov/protein/373159252?report=genbank&log$=prottop&blast_rank=5&RID=) |
| Arabian oryx (isolate SAZ07) | *Oryx leucoryx* | [JQ811847.1](http://www.ncbi.nlm.nih.gov/nuccore/403330310) | [AFR42406.1](http://www.ncbi.nlm.nih.gov/protein/403330311) |
| Banteng | *Bos javanicus* | [JQ811841.1](http://www.ncbi.nlm.nih.gov/nuccore/403330298) | [AFR42400.1](http://www.ncbi.nlm.nih.gov/protein/403330299) |
| Black Flying Fox (type of bat) | *Pteropus alectogi* | [GU045604.1](http://www.ncbi.nlm.nih.gov/nuccore/308055167) | [ADO01607.1](http://www.ncbi.nlm.nih.gov/protein/308055168) |
| Bottlenose dolphin | *Tursiops truncatus* | [XM_004331556.1](http://www.ncbi.nlm.nih.gov/nuccore/470659827) | [XP_004331604.1](http://www.ncbi.nlm.nih.gov/protein/470659828) |
| Cat | *Felis catus* | [XM_004001330.1](http://www.ncbi.nlm.nih.gov/nuccore/410985668) | [XP_004001379.1](http://www.ncbi.nlm.nih.gov/protein/410985669) |
| Chamois | *Rupicapra rupicapra* | [JQ811845.1](http://www.ncbi.nlm.nih.gov/nuccore/403330306) | [AFR42404.1](http://www.ncbi.nlm.nih.gov/protein/403330307) |
| Chicken | *Gallus gallus* | [NM_001024586.1](http://www.ncbi.nlm.nih.gov/nuccore/66793458) | [NP_001019757.1](http://www.ncbi.nlm.nih.gov/entrez/viewer.fcgi?db=protein&val=66793459&itool=HomoloGeneMainReport) |
| Common marmoset (primate) | *Callithrix jacchus* | [XM_002760480.2](http://www.ncbi.nlm.nih.gov/nuccore/390477235) | [XP_002760526.1](http://www.ncbi.nlm.nih.gov/protein/296230015) |
| Cow (Holstein) | *Bos taurus* | [DQ335128.1](https://www.ncbi.nlm.nih.gov/nuccore/84874623) | [ABC68311.1](http://www.ncbi.nlm.nih.gov/protein/84874624) |
| Dog | *Canis lupus familiaris* | [NM_001197176.1](http://www.ncbi.nlm.nih.gov/nuccore/308210836) | [NP_001184105.1](http://www.ncbi.nlm.nih.gov/entrez/viewer.fcgi?db=protein&val=308210837&itool=HomoloGeneMainReport) |
| Elephant African | *Loxodonta africana* | [XM_010591125.1](http://www.ncbi.nlm.nih.gov/nuccore/731482296) | [XP_010589427.1](http://www.ncbi.nlm.nih.gov/protein/731482297) |
| Ferret | *Mustela putorius furo* | [ENSMPUT00000018908](http://www.ensembl.org/Mustela_putorius_furo/Transcript/Summary?db=core;g=ENSMPUG00000018756;r=GL897329.1:120501-123077;t=ENSMPUT00000018908) | [ENSMPUP00000018636](http://www.ensembl.org/Mustela_putorius_furo/Transcript/ProteinSummary?db=core;g=ENSMPUG00000018756;r=GL897329.1:120501-123077;t=ENSMPUT00000018908) |
| Florida Manatee | *Trichechus manatus latirostris* | [XM_004375953.1](http://www.ncbi.nlm.nih.gov/nuccore/471371000) | [XP_004376010.1](http://www.ncbi.nlm.nih.gov/protein/471371001) |
| Fresh water Yangtze River dolphin | *Lipotes vexillifer* | [XM_007450222.1](http://www.ncbi.nlm.nih.gov/nucleotide/602729725?report=genbank&log$=nuclalign&blast_rank=15&RID=1KC4R0HK015) | [XP_007450284.1](http://www.ncbi.nlm.nih.gov/protein/602729726) |
| Gaur (domesticated: gayal or mithun) | *Bos frontalis* | [KF905601.1](http://www.ncbi.nlm.nih.gov/nuccore/641399287) | [AIA59656.1](http://www.ncbi.nlm.nih.gov/protein/641399288) |
| gemsbok or gemsbuck (antelope) | *Oryx gazella* | [JQ811846.1](http://www.ncbi.nlm.nih.gov/nuccore/403330308) | [AFR42405.1](http://www.ncbi.nlm.nih.gov/protein/403330309) |
| Giant Panda | *Ailuropoda melanoleuca* | [ENSAMET00000022014](http://www.ensembl.org/Ailuropoda_melanoleuca/Transcript/Summary?db=core;g=ENSAMEG00000020191;r=GL192912.1:262041-264617;t=ENSAMET00000022014) | [ENSAMEP00000021245](http://www.ensembl.org/Ailuropoda_melanoleuca/Transcript/ProteinSummary?db=core;g=ENSAMEG00000020191;r=GL192912.1:262041-264617;t=ENSAMET00000022014) |
| Giraffe (isolate KB3332) | *Giraffa camelopardalis* | [JQ811844.1](http://www.ncbi.nlm.nih.gov/nuccore/403330304) | [AFR42403.1](http://www.ncbi.nlm.nih.gov/protein/403330305) |
| Goat | *Capra hircus* | [HQ188378.1](http://www.ncbi.nlm.nih.gov/nuccore/321171049) | [ADW76745.1](http://www.ncbi.nlm.nih.gov/protein/321171050?report=genbank&log$=prottop&blast_rank=7&RID=) |
| Golden mole | *Chrysochloris asiatica* | [XM_006871148.1](http://www.ncbi.nlm.nih.gov/nuccore/586481380) | [XP_006871210.1](http://www.ncbi.nlm.nih.gov/protein/586481381) |
| Gorilla | *Gorilla gorilla* | [AB445648.1](http://www.ncbi.nlm.nih.gov/nuccore/194068448) | [BAG55045.1](http://www.ncbi.nlm.nih.gov/protein/194068449) |
| Greater kudu (SAZ15) | *Tragelaphus strepsiceros* | [JQ811848.1](http://www.ncbi.nlm.nih.gov/nuccore/403330312) | [AFR42407.1](http://www.ncbi.nlm.nih.gov/protein/403330313) |
| Grey tailed opossum (marsupial) | *Monodelphis domestica* | [XM_001376152](http://www.metalife.com/SearchSummary.aspx?query=%5BAccession%5DXM_001376152&db=Refseq Nucleic) | [XP_001376189.1](http://www.metalife.com/SearchSummary.aspx?query=%5BVersion%5DXP_001376189.1&db=Refseq Protein) |
| Hedgehog | *Erinaceus europaeus* | [XM_007530177.1](http://www.ncbi.nlm.nih.gov/nuccore/617633852) | [XP_007530239.1](http://www.ncbi.nlm.nih.gov/protein/617633853) |
| Horse | *Equus callabri* | Horse (unpub*) | Horse (unpub*) |
| Human | *Homo sapiens* | [NM_003268.5](http://www.ncbi.nlm.nih.gov/nuccore/281427130) | [NP_003259.2](http://www.ncbi.nlm.nih.gov/protein/16751843) |
| Killer whale | *Orcinus orca* | [XM_004271004.1](http://www.ncbi.nlm.nih.gov/nuccore/466015071) | [XP_004271052.1](http://www.ncbi.nlm.nih.gov/protein/466015072) |
| Lesser kudu (isolate SAZ17) | *Tragelaphus imberbis* | [JQ811849.1](http://www.ncbi.nlm.nih.gov/nuccore/403330314) | [AFR42408.1](http://www.ncbi.nlm.nih.gov/protein/403330315) |
| Mouse | *Mus musculus* | [NM_016928.2](http://www.ncbi.nlm.nih.gov/nuccore/124248589) | [NP_058624.2](http://www.ncbi.nlm.nih.gov/entrez/viewer.fcgi?db=protein&val=124248590&itool=HomoloGeneMainReport) |
| Mule deer (isolate JEW01) | *Odocoileus hemionus* | [JQ811842.1](http://www.ncbi.nlm.nih.gov/nuccore/403330300) | [AFR42401.1](http://www.ncbi.nlm.nih.gov/protein/403330301) |
| Nine-banded armadillo | *Dasypus novemcinctus* | [XM_004471726.2](http://www.ncbi.nlm.nih.gov/nuccore/821128816) | [XP_004471783.1](http://www.ncbi.nlm.nih.gov/protein/488564179) |
| Phillipine Tarsier (primate) | *Tarsius syrichta* | [ENSTSYT00000011896](http://www.ensembl.org/Tarsius_syrichta/Transcript/Summary?db=core;g=ENSTSYG00000011910;r=scaffold_22642:608-3187;t=ENSTSYT00000011896) | [ENSTSYP00000010915](http://www.ensembl.org/Tarsius_syrichta/Transcript/ProteinSummary?db=core;g=ENSTSYG00000011910;r=scaffold_22642:608-3187;t=ENSTSYT00000011896) |
| Pig | *Sus scrofa* | [KF019633.1](http://www.ncbi.nlm.nih.gov/nuccore/373159247) | [AGT79978.1](http://www.ncbi.nlm.nih.gov/protein/373159248?report=genbank&log$=prottop&blast_rank=3&RID=) |
| Pika | *Ochotona princeps* | [ENSOPRT00000013962](http://www.ensembl.org/Ochotona_princeps/Transcript/Summary?db=core;g=ENSOPRG00000013976;r=GeneScaffold_2645:500765-503341;t=ENSOPRT00000013962) | [ENSOPRP00000012743](http://www.ensembl.org/Ochotona_princeps/Transcript/ProteinSummary?db=core;g=ENSOPRG00000013976;r=GeneScaffold_2645:500765-503341;t=ENSOPRT00000013962) |
| Platypus | *Ornithorhynchus anatinus* | [XM_001512183.3](http://www.ncbi.nlm.nih.gov/nuccore/XM_001512183.3) | [XP_001512233.3](http://www.ncbi.nlm.nih.gov/protein/XP_001512233.3) |
| Przewalski's horse | *Equus ferus przewalskii* | [XM_008533391.1](http://www.ncbi.nlm.nih.gov/nuccore/664748448) | [XP_008531613.1](http://www.ncbi.nlm.nih.gov/protein/664748449) |
| Rabbit | *Oryctolagus cuniculus* | [HQ874605.1](http://www.ncbi.nlm.nih.gov/nuccore/655860040) | [AEA11027.1](http://www.ncbi.nlm.nih.gov/protein/655860041) |
| Rat | *Rattus norvegicus* | [NM_001145828.1](http://www.ncbi.nlm.nih.gov/nuccore/224967051) | [NP_001139300.1](http://www.ncbi.nlm.nih.gov/protein/224967052) |
| Rhinoceros | *Ceratotherium simum simum* | [XM_004439536.1](http://www.ncbi.nlm.nih.gov/nuccore/478530873) | [XP_004439593.1](http://www.ncbi.nlm.nih.gov/protein/478530874) |
| Roe Deer | *Capreolus capreolus* | [KM488255](http://www.ebi.ac.uk/ena/data/view/KM488255) | [AIZ77221.1](http://www.ncbi.nlm.nih.gov/protein/732663988) |
| Scammon's minke whale | *Balaenoptera acutorostrata scammoni* | [XM_007172037.1](http://www.ncbi.nlm.nih.gov/nuccore/XM_007172037.1) | [XP_007172099.1](http://www.ncbi.nlm.nih.gov/protein/XP_007172099.1) |
| Scimitar oryx isolate SAZ21 | *Oryx dammah* | [JQ811850.1](http://www.ncbi.nlm.nih.gov/nuccore/403330316) | [AFR42409.1](http://www.ncbi.nlm.nih.gov/protein/403330317) |
| Sheep | *Ovis aries* | [NM_001135926.1](http://www.ncbi.nlm.nih.gov/nuccore/209693401) | [NP_001129398.1](http://www.ncbi.nlm.nih.gov/protein/209693402?report=genbank&log$=prottop&blast_rank=8&RID=) |
| Sloth | *Choloepus hoffmanni* | [ENSCHOT00000011335](http://www.ensembl.org/Choloepus_hoffmanni/Transcript/Summary?db=core;g=ENSCHOG00000011312;r=scaffold_132520:2067-4729;t=ENSCHOT00000011335) | [ENSCHOP00000009998](http://www.ensembl.org/Choloepus_hoffmanni/Transcript/ProteinSummary?db=core;g=ENSCHOG00000011312;r=scaffold_132520:2067-4729;t=ENSCHOT00000011335) |
| Sperm whale (transcript variant X5) | *Physeter catodon (or macrocephalus)* | [XM_007121467.1](http://www.ncbi.nlm.nih.gov/nuccore/593765984) | [XP_007121529.1](http://www.ncbi.nlm.nih.gov/protein/593765985) |
| Tenrec (lesser hedgehog) | *Echinops telfairi* | [ENSETET00000013405](http://www.ensembl.org/Echinops_telfairi/Transcript/Summary?db=core;g=ENSETEG00000013406;r=scaffold_216931:2145-4721;t=ENSETET00000013405) | [ENSETEP00000010870](http://www.ensembl.org/Echinops_telfairi/Transcript/ProteinSummary?db=core;g=ENSETEG00000013406;r=scaffold_216931:2145-4721;t=ENSETET00000013405) |
| Tibetan antelope | *Pantholops hodgsonii* | [XM_005962090.1](http://www.ncbi.nlm.nih.gov/nuccore/556732991) | [XP_005962152.1](http://www.ncbi.nlm.nih.gov/protein/556732992) |
| Water or River Buffalo | *Bubalus bubalis* | [JN615231.1](http://www.ncbi.nlm.nih.gov/nuccore/373159241) | [AEY63772.1](http://www.ncbi.nlm.nih.gov/protein/373159242) |
| White-tailed deer (isolate JEW21) | *Odocoileus virginianus* | [JQ811843.1](http://www.ncbi.nlm.nih.gov/nuccore/403330302) | [AFR42402.1](http://www.ncbi.nlm.nih.gov/protein/403330303) |
| wild Bactrian Camel | *Camelus ferus* | [XM_006188136.1](http://www.ncbi.nlm.nih.gov/nuccore/560924012) | [XP_006188198.1](http://www.ncbi.nlm.nih.gov/protein/560924013) |
| Yak | *Bos grunniens* | [GU647093.1](http://www.ncbi.nlm.nih.gov/nuccore/308157263) | [ADO15559.1](http://www.ncbi.nlm.nih.gov/protein/308157264?report=genbank&log$=prottop&blast_rank=4&RID=) |
| Zebra Fish | *Danio rerio* | [NM_001130595.1](http://www.ncbi.nlm.nih.gov/nuccore/194578862) | [NP_001124067.1](http://www.ncbi.nlm.nih.gov/protein/194578863) |
| Zebu(Sahiwal) | *Bos indicus* | [GQ248711.1](http://www.ncbi.nlm.nih.gov/nuccore/294719803) | [ADF32904.1](http://www.ncbi.nlm.nih.gov/protein/294719804) |

*Kindly provided by Dr James MacLeod, University of Kentucky, USA.

**Supplementary Table S2 Summary of siRNA used for target gene knock-down.**

|  |  |  |  |  |
| --- | --- | --- | --- | --- |
| Gene | Species | Accession No. | Start site | siRNA Name/Sequence 5’-3’ |
|  |  |  |  |  |
|  |  |  |  |  |
| mitogen-activated protein kinase 14 | Human | NM_001315 | 784 | SASI_Hs01_00018464 |
| (MAPK14/p38) | Bovine | NM_001102174 | 1161 | GCAAGAAACTACATTCAGT |
|  |  |  |  |  |
| v-rel avian reticuloendotheliosis viral | Human | NM_021975 | 420 | SASI_Hs01_00171091 |
| oncogene homolog A (RELA) | Bovine | NM_001080242 | 263 | CTATCAAGATCAATGGCTA |
|  |  |  |  |  |
| TNF receptor-associated factor 6, E3 | Human | NM_145803 | 770 | SASI_Hs01_00224533 |
| ubiquitin protein ligase (TRAF6) | Bovine | NM_001034661 | 981 | GAGAATACCCAGTCGCATA |
|  |  |  |  |  |
| phosphoinositide-3-kinase, regulatory subunit | Human | NM_181523 | 1599 | SASI_Hs01_00022804 |
| 1 (alpha) (PIK3R1) | Bovine | NM_174575 | 1114 | PIK3R1#b1 CTAAGGAAAGGAGGAAATA |
|  | Bovine | NM_174575 | 676 | PIK3R1#b2 CTCATTAGGTCGCCCAGCA |
|  | Bovine | NM_174575 | 1456 | PIK3R1#b3 GCATTTAATGAAACCATAA |
|  |  |  |  |  |

The human siRNA are proprietary (Sigma-Alrich) and therefore only the approximate start site of the sequence they align to is available.

**Supplementary Table S3 Oligonucleotides used for RT-qPCR analysis of target gene knock-down.**

|  |  |  |  |  |
| --- | --- | --- | --- | --- |
| Gene | Species | Accession No. | Orientation | Sequence 5’-3’ |
|  |  |  |  |  |
|  |  |  |  |  |
| mitogen-activated protein kinase 14 | Human | NM_001315 | F | AGATTCTGGATTTTGGACTG |
| (p38/MAPK14) |  |  | R | ACAGTTGCCATGTAGACC |
|  | Bovine | NM_001102174 | F | CTGATGCTAAGAGCGGGTGA |
|  |  |  | R | CAGATGGCAAGAATGGAAATG |
| v-rel avian reticuloendotheliosis viral oncogene | Human | NM_021975 | F | GCAGAAAGAGGACATTGAG |
| homolog A (RELA) |  |  | R | GTGCACATCAGCTTGC |
|  | Bovine | NM_001080242 | F | AAGCCCTCCCAAAGCACA |
|  |  |  | R | CCAGGAAGACATCACCCAAG |
| TNF receptor-associated factor 6, E3 ubiquitin | Human | NM_145803 | F | TACTCATCAGAGAACAGATGC |
| protein ligase (TRAF6) |  |  | R | AAAGTACTGAATGTGCATGG |
|  | Bovine | NM_001034661 | F | GTGAAAACAGCTGTGGATCCAGC |
|  |  |  | R | GCTTTCCAGGGGTGGGTCAAAT |
| phosphoinositide-3-kinase, regulatory subunit 1 | Human | NM_181523 | F | AAGCAGCAACCGAAACAAAG |
| (alpha) (PIK3R1) |  |  | R | CGTCCACCACTACAGAGCAG |
|  | Bovine | NM_174575 | F | GCAGACAGTGAGCAACAAGC |
|  |  |  | R | GATAAGAAGCGGTGGAGCAA |
| chromosome alignment maintaining phosphoprotein | Human | NM_032436 | F | GACAGCAGCAGTATTGAAAG |
| 1 (CHAMP1) |  |  | R | GTTTACGAAGTTCCTGGAATG |
|  | Bovine | NM_001205506 | F | AGCAGTGACCAAGAGCAGGT |
|  |  |  | R | TCATAGCACGACAGCAACAA |
|  |  |  |  |  |

Oligonucleotides for human p38, RELA, TRAF6 and CHAMP1 were predesigned and validated by Sigma-Aldrich. F and R denote forward and reverse primers respectively.
